# Mechanical Effects of Cellulose, Xyloglucan, and Pectins on Stomatal Guard Cells of *Arabidopsis thaliana*

**DOI:** 10.3389/fpls.2018.01566

**Published:** 2018-11-05

**Authors:** Hojae Yi, Yue Rui, Baris Kandemir, James Z. Wang, Charles T. Anderson, Virendra M. Puri

**Affiliations:** ^1^Department of Agricultural and Biological Engineering, The Pennsylvania State University, University Park, PA, United States; ^2^Department of Biology, The Pennsylvania State University, University Park, PA, United States; ^3^Intercollege Graduate Degree Program in Plant Biology, The Pennsylvania State University, University Park, PA, United States; ^4^College of Information Sciences and Technology, The Pennsylvania State University, University Park, PA, United States

**Keywords:** stomatal guard cell, plant cell wall, stomatal mechanics, finite element modeling, confocal microscopy, image analysis

## Abstract

Stomata function as osmotically tunable pores that facilitate gas exchange at the surface of plants. Stomatal opening and closure are regulated by turgor changes in guard cells that result in mechanically regulated deformations of guard cell walls. However, how the molecular, architectural, and mechanical heterogeneities that exist in guard cell walls affect stomatal dynamics is unclear. In this work, stomata of wild type *Arabidopsis thaliana* plants or of mutants lacking normal cellulose, hemicellulose, or pectins were experimentally induced to close or open. Three-dimensional images of these stomatal complexes were collected using confocal microscopy, images were landmarked, and three-dimensional finite element models (FEMs) were constructed for each complex. Stomatal opening was simulated with a 5 MPa turgor increase. By comparing experimentally measured and computationally modeled changes in stomatal geometry across genotypes, anisotropic mechanical properties of guard cell walls were determined and mapped to cell wall components. Deficiencies in cellulose or hemicellulose were both predicted to stiffen guard cell walls, but differentially affected stomatal pore area and the degree of stomatal opening. Additionally, reducing pectin molecular mass altered the anisotropy of calculated shear moduli in guard cell walls and enhanced stomatal opening. Based on the unique architecture of guard cell walls and our modeled changes in their mechanical properties in cell wall mutants, we discuss how each polysaccharide class contributes to wall architecture and mechanics in guard cells. This study provides new insights into how the walls of guard cells are constructed to meet the mechanical requirements of stomatal dynamics.

## Introduction

Stomata function as osmotically tunable pores that control CO_2_ intake and water loss at the surface of plants. Each stomatal pore is surrounded by a pair of specialized guard cells, which gain turgor to pressurize and open the pore, and lose turgor to depressurize and close the pore (Meidner and Mansfield, 1968; Aylor et al., 1973; Zeiger et al., 1987; Niklas, 1992; Franks et al., 1998; Franks and Farquhar, 2001, 2007). For plants to efficiently control photosynthesis and transpiration by stomatal opening and closure, stomatal guard cells must repeatedly expand and contract. Thus, the reversible deformations of guard cells make them an intriguing model to study the elastic mechanics of complex biomaterials, complementing studies of cell walls that undergo structural and compositional changes during inelastic growth in other plant cell types (Cosgrove, 2005).

Stomatal opening is thought to be driven by the anisotropic deformation of the guard cell, and such anisotropy is hypothesized to be linked to the molecular construction and mechanical properties of the guard cell wall (Meckel et al., 2007; Amsbury et al., 2016; Rui and Anderson, 2016; Carter et al., 2017; Marom et al., 2017; Woolfenden et al., 2017). For example, cellulose microfibrils (CMFs) have high stiffness, presumably preventing circumferential expansion and favoring cell elongation during stomatal opening (Meckel et al., 2007). Stomata in cellulose-deficient *cesa3*^*je*5^ mutants have larger pore widths and less anisotropic cellulose organization in the closed state (Rui and Anderson, 2016). The role of hemicellulose, namely xyloglucan in *Arabidopsis*, has been highlighted by the finding that stomata of *xxt1 xxt2* mutants lacking xyloglucan exhibit smaller pore widths in both open and closed states (Rui and Anderson, 2016). Several reports have found evidence for the role of pectins in controlling the elasticity of guard cell walls and the dynamic range of stomata (Jones et al., 2003, 2005; Amsbury et al., 2016; Rui et al., 2017).

Despite extensive investigations of stomatal development (Pillitteri and Torii, 2012) and physiology (Kim et al., 2010), the precise relationships between the structure and composition of guard cell walls and the mechanical function of stomata remain elusive. The mechanics of the plant cell wall can be described by a set of constitutive laws linking extrinsic forces on the wall and its resulting deformation. Hooke's law provides a coherent approach to modeling the elastic behavior of guard cells, i.e., their reversible expansion that disappears when force is removed (DeMichele and Sharpe, 1973; Edwards et al., 1976; Sharpe and Wu, 1978; Franks et al., 1998). To apply Hooke's law to an object with complex geometry and anisotropic mechanical properties, as is the case for guard cell walls, numerical methods should be employed. In previous studies, guard cell shape and dynamics have been modeled using finite element modeling (FEM) (Bathe, 1996; Zienkiewicz et al., 2014) albeit with idealized geometries (Cooke et al., 1976; Wu and Sharpe, 1979; Marom et al., 2017; Woolfenden et al., 2017). Thus, further work is needed to connect the geometries of real stomatal complexes and modeled wall mechanics with stomatal dynamics, in genotypes with normal or altered cell walls.

**Figure 1:**
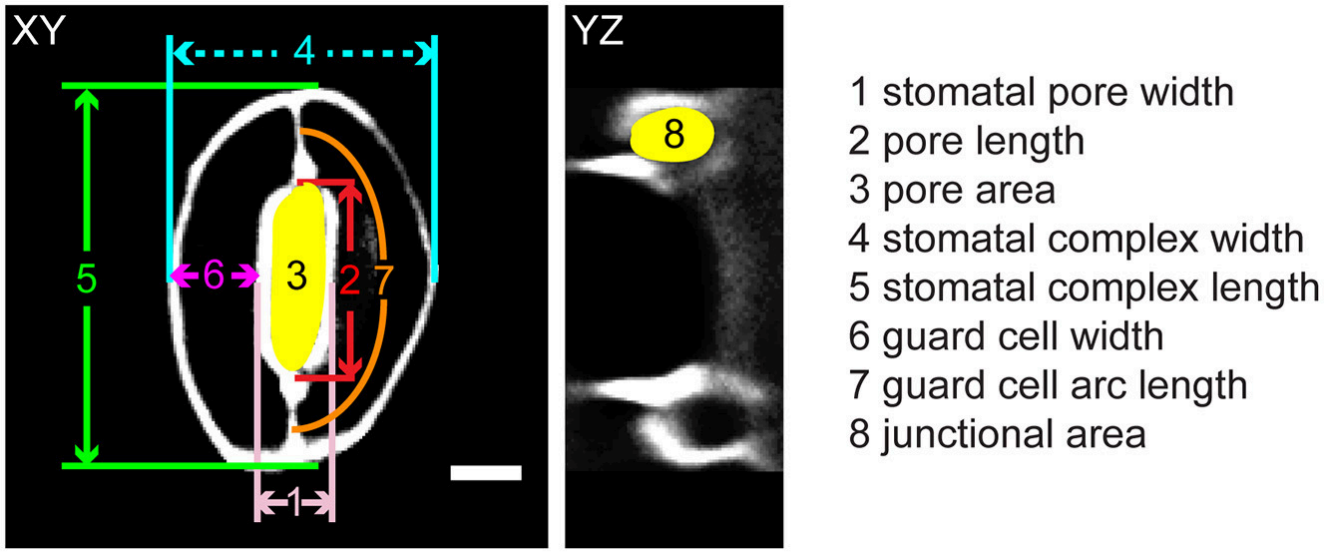
Legend of measurements in a stomatal complex from 3D confocal imaging of propidium iodide-stained samples. Scale bar is 5 μm.

Here, we examined the contributions of cellulose, xyloglucan, and pectins to the dynamics and mechanical properties of stomatal guard cells of *Arabidopsis thaliana*. To (1) minimize the effects of idealized geometric assumptions on boundary conditions describing constraints on a stomatal complex, (2) accurately account for the degree of freedom at the stomatal junction area, and (3) pinpoint locations and areas where guard cells interact with neighboring cells, we modeled stomatal guard cells using the contours of actual stomata by computationally tracing 3D confocal images of guard cells and inputting these coordinates directly into our FEMs. We created FEMs of stomatal complexes that recapitulate the geometries and dynamics of wild type (Columbia, Col-0) *Arabidopsis* plants, and three mutant *Arabidopsis* lines: *cesa3*^*je*5^, which is defective in cellulose synthesis (Desprez et al., 2007); *xxt1 xxt2*, which lacks the hemicellulose, xyloglucan (Cavalier et al., 2008; Park and Cosgrove, 2012); and *PGX1 OE*, which overexpresses *POLYGALACTURONASE INVOLVED IN EXPANSION1* (*PGX1*) and has pectic homogalacturonan (HG) with a smaller average molecular mass (Xiao et al., 2014). In these FEMs, guard cell walls were modeled as geometrically continuous shells of varying thickness with anisotropic mechanical properties. Through simulation of stomatal opening via turgor pressure increase, we identified sets of anisotropic mechanical properties in our FEMs that match observed stomatal geometries in the open state, including stomatal pore width, stomatal complex length, and guard cell width (Figure 1). By comparing anisotropic stiffness coefficients of guard cell walls in different genotypes with knowledge of their molecular composition and structures, this study reveals potential mechanisms by which major wall polysaccharides contribute to the mechanical regulation of stomatal opening and closure.

## Materials and methods

### Plant materials and growth conditions

*Arabidopsis thaliana* seeds of the Col-0 ecotype, and mutants *cesa3*^*je*5^ (Desprez et al., 2007), *xxt1 xxt2* (Arabidopsis Biological Resource Center stock no. CS16349) (Cavalier et al., 2008), and *PGX1 OE* (Xiao et al., 2014) were surface sterilized in 30% bleach with 0.1% SDS for 20 min, washed in sterile water four times, and stored in 0.15% agar at 4°C for at least 2 d for stratification before sowing on $\frac{1}{2}$ MS plates (2.2 g/L Murashige and Skoog salts, 0.6 g/L MES, pH 5.6) containing 1% w/v sucrose and germinating at 22°C under 24 h illumination in a Percival CU36-L5 growth chamber. Ten-d-old seedlings were transferred from plates to Fafard C2 Soil supplemented with Miracle-Gro and grown at 22°C under 16 h light/8 h dark conditions.

### Estimation of guard cell wall thickness

Trimming, fixation, serial dehydration, LR White infiltration and polymerization were performed as described in Amsbury et al. (2016). Two μm-thick sections of each leaf sample were cut on a Leica UC6 ultramicrotome (Buffalo Grove, IL) with a glass knife. Sections were stained with 0.05% toluidine blue for 10–30 s and rinsed with water to remove excess toluidine blue. Sections were then imaged with the transmission light on a Zeiss Axio Observer microscope with a 100X 1.4 numerical aperture immersion oil objective and a Nikon D5100 DSLR camera. Images were analyzed in ImageJ. Because guard cell walls are differentially thickened (Zhao and Sack, 1999), wall thickness was measured at five different regions for a given guard cell, including the lower periclinal wall, the upper periclinal wall at cuticular ledges, the upper periclinal wall away from cuticular ledges, the ventral wall, and the dorsal wall. Representative images of toluidine blue-stained cross sections of guard cells are presented in Supplemental Figure 1, and measurements of guard cell wall thickness at these regions are presented in Supplemental Table 1.

### Propidium iodide staining and confocal microscopy

Rosette leaves were collected from 3- to 4-week-old plants. Stomatal opening was induced by incubating leaves in a buffer containing 20 mM KCl, 1 mM CaCl_2_, and 5 mM MES-KOH, pH 6.15, in light at 22°C in a Percival CU36-L5 growth chamber for 2.5 h. Stomatal closure was induced by incubating leaves in a buffer containing 50 mM KCl, 0.1 mM CaCl_2_, and 10 mM MES-KOH, pH 6.15, in the dark at 22°C in a Percival CU36-L5 growth chamber for 2.5 h. Leaves were then stained with 100 μg/ml propidium iodide (PI, Life Technologies; catalog no. P3566) for 5 min before imaging. Z-stack images were collected on a Zeiss Axio Observer microscope with a Yokogawa CSU-X1 spinning disk head and a 63X 1.4 numerical aperture immersion oil objective, using a 561 nm excitation laser and a 617/73 nm emission filter with a step size of 0.2 μm. Z-stack images were subjected to 3D blind deconvolution using AutoQuant X2 (Media Cybernetics) software.

### Semi-computerized geometry measurements of guard cells and stomatal pores

A semi-computerized active contours-based method was adopted to segment and measure the pore and cell-pair areas (Kass et al., 1988). In this approach, the user initializes a closed curve by entering a few points. The initialized curve expands and evolves according to the edge map of the image, fitting to the irregularly shaped object. The evolution is driven by an optimization scheme where the cost is minimized when the curve fits the edges smoothly.

Pore area values across each z–stack were obtained via the aforementioned method. To account for outliers due to contrast issues, a robust nonlinear regression (fourth-order polynomial) was applied on the area profile graph, where the variance was assumed to have a Cauchy distribution (Motulsky and Brown, 2006). The minimum of the regression curve was picked as the pore area in number of pixels. As the physical area of a pixel in the XY plane is 0.0409 μm^2^, the pore area in pixels was multiplied by this number to obtain the actual area. A connected component analysis was carried out for minimum pore area segmentation. The major and minor axes of the connected component were computed to measure the height and the width of the pore area, respectively.

A similar analysis was administered for cell geometry measurements. Active contours segmentation was carried out to obtain stomatal complex length and width in the image slice that contained the minimum pore area, or another slice within a four-slice neighborhood, where contrast for guard cell perimeters was clearer. The same connected component analysis was conducted to measure stomatal complex length and width.

After obtaining binary masks for cell and pore area, the tangential length of each cell was computed. For this task, intensity profile analysis in the radial direction originating from the center of mass for the pore opening was conducted at each angle within the interval [0°, 359°]. The local intensity maxima representing pore and cell borders were detected and the midpoint of the line segment between the two points was calculated. This method provided a medial axis for each cell within the pair. The medial axis was smoothed through the Savitzky-Golay filter, where subsets of data were re-fit via polynomial least squares (Savitzky and Golay, 1964). The medial axis curves were simplified through polynomial approximation to minimize meandering and jitter. The arc length of the simplified medial axis was computed to measure the tangential length of individual cells.

### Construction of finite element models of guard cell pairs

**Figure 2:**
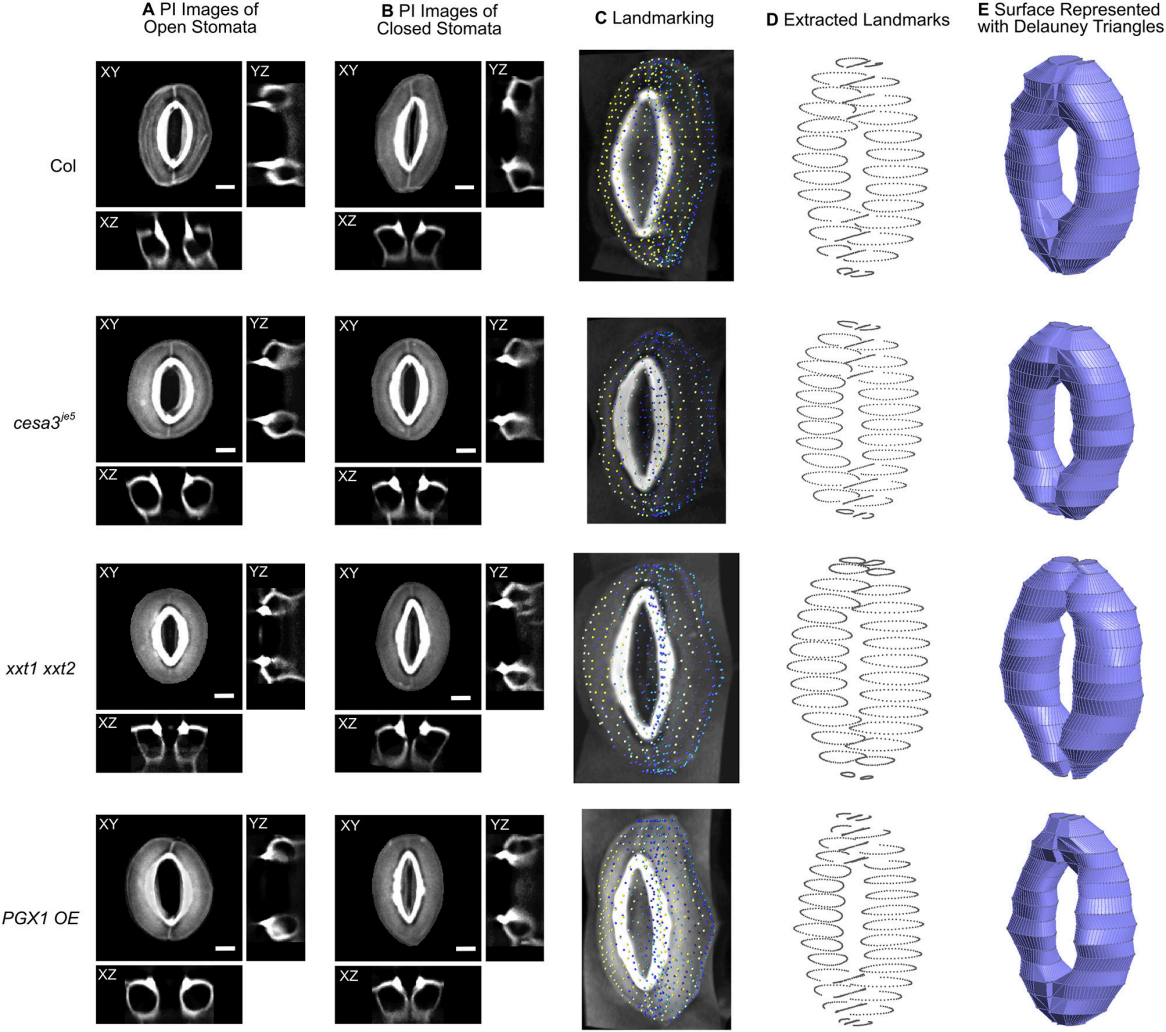
Representative 3D images of open and closed stomata and representative FE models of closed stomata in Col-0, *cesa3*^*je*5^, *xxt1 xxt2*, and *PGX1 OE* plants. **(A)** Representative micrographs of typical stomata in the open state taken from 3-D confocal imaging of propidium iodide (PI)-stained stomatal guard cells, with XY, XZ, and YZ projections; surrounding pavement cells were digitally cropped. **(B)** Confocal micrographs of stomata in the closed state. Scale bars represent 5 μm. **(C–E)** An illustration of the procedure of developing surface models of closed guard cells of Col-0, *cesa3*^*je*5^, *xxt1 xxt2*, and *PGX1 OE* genotypes for finite element (FE) modeling of stomatal opening. **(C)** Landmarks on cropped z-stacked images made by manually tracing guard cell walls using Vaa3d (Peng et al., 2010). **(D)** Extracted landmarks representing the middle plane of the guard cell walls. These landmarks were used to develop surfaces meshed as Delauney triangles. **(E)** Construction of wireframe and surface models was performed using Gmsh 2.14 (Geuzaine and Remacle, 2009). Ten surface models were generated for each genotype (Supplemental Figure 2) and were further developed into finite element (FE) models.

Three-dimensional representations of guard cells were generated by tracing the midpoints of guard cell walls from 3-week-old rosette leaves in Col-0, *cesa3*^*je*5^, *xxt1 xxt2*, and *PGX1 OE* plants stained with PI to label cell walls. Z-stack images of stomatal complexes in the closed state induced by dark treatment were imported into Vaa3d (Peng et al., 2010) to be traced and landmarked along the middle plane of the guard cell wall (Figure 2C). To ensure the smoothness of the eventual model, traced landmarks were fed to create parameterized B-spline representations using “*splprep*” (Dierckx, 1982) in the NumPy library (Jones et al., 2001). Three-dimensional vertices were extracted from the B-spline curve to produce loop elements representing segments of a guard cell wall. Finally, this information was processed to produce triangular elements using Gmsh 2.14 (Geuzaine and Remacle, 2009) as illustrated in Figure 2D. These triangular elements were used to develop finite element models of stomatal complexes that are constructed with shell elements, including mechanical interactions with neighboring cells, using Abaqus (Dassault Systèmes, 2016). The triangular, three-node conventional stress/displacement reduced-integration element “S3R” was used to calculate changes in guard cell wall under applied turgor pressures (Dassault Systèmes, 2016). Interactions with pavement cells were modeled as displacement boundary conditions using experimentally observed changes in stomatal complex length and stomatal complex width (Figure 1 and Table 1). These prescribed boundary conditions resulted in additional loading on stomatal junction areas and dorsal areas, respectively.

**Table 1:** Measurement of stomatal pore dimensions, guard cell pair dimensions, guard cell junction area, and guard cell geometry in wild type (Col-0), *cesa3*^*je*5^, *xxt1 xxt2*, and *PGX1 OE* plants from 3D imaging.

**Treatment**	**Genotype**	**Avg stomatal pore width (μm)**	**Avg stomatal pore length (μm)**	**Avg aspect ratio of pores**	**Avg pore area (μm^2^)**	**Avg stomatal complex length (μm)**	**Avg stomatal complex width (μm)**	**Avg guard cell junction area (μm^2^)**	**Avg guard cell width (μm)**	**Avg guard cell arc length (μm)**
darkness (closed)	Col-0	1.2 ± 0.1^a^	11.9 ± 0.4^a^	0.10 ± 0.01^a^	12.6 ± 1.1^a^	24.5 ± 0.5^ab^	14.8 ± 0.3^a^	49.9 ± 1.6^a^	6.5 ± 0.2^a^	20.7 ± 0.4^a^
	*cesa3^*je*5^*	2.5 ± 0.2^b^	11.2 ± 0.4^a^	0.23 ± 0.01^b^	24.6 ± 1.6^b^	22.9 ± 0.5^a^	15.2 ± 0.4^a^	46.6 ± 1.3^a^	6.2 ± 0.2^a^	22.6 ± 0.6^bc^
	*xxt1 xxt2*	0.6 ± 0.1^c^	12.2 ± 0.4^a^	0.05 ± 0.01^c^	7.1 ± 1.0^c^	24.8 ± 0.5^b^	14.4 ± 0.2^a^	46.1 ± 0.9^a^	6.3 ± 0.2^a^	19.7 ± 0.3^ac^
	*PGX1 OE*	0.7 ± 0.1^c^	12.4 ± 0.3^a^	0.06 ± 0.01^c^	10.3 ± 1.2^ac^	25.1 ± 0.4^b^	13.0 ± 0.3^b^	46.4 ± 1.0^a^	5.9 ± 0.1^a^	22.2 ± 1.3^a^
light(open)	Col-0	3.9 ± 0.2^a^	13.6 ± 0.5^a^	0.30 ± 0.02^ac^	42.3 ± 2.6^a^	25.4 ± 0.5^a^	16.9 ± 0.3^a^	44.1 ± 0.9^a^	6.5 ± 0.1^a^	24.0 ± 0.3^a^
	*cesa3^*je*5^*	4.7 ± 0.4^a^	10.0 ± 0.9^bc^	0.48 ± 0.03^b^	41.8 ± 4.8^a^	22.7 ± 0.5^bc^	18.1 ± 0.3^a^	48.0 ± 0.9^b^	6.7 ± 0.1^a^	23.3 ± 0.5^a^
	*xxt1 xxt2*	2.3 ± 0.2^b^	8.5 ± 0.6^c^	0.29 ± 0.02^c^	17.5 ± 2.0^b^	21.8 ± 0.5^b^	15.2 ± 0.2^b^	45.2 ± 1.0^ab^	6.5 ± 0.1^a^	20.0 ± 0.3^a^
	*PGX1 OE*	4.5 ± 0.2^a^	12.0 ± 0.5^ab^	0.38 ± 0.02^a^	45.6 ± 3.7^a^	24.3 ± 0.5^ac^	18.0 ± 0.4^a^	45.6 ± 1.0^ab^	6.8 ± 0.1^a^	24.4 ± 0.5^b^

*Stomatal pore width, pore length, aspect ratio of pores (width/length), pore area, stomatal complex length, stomatal complex width, guard cell junction area, guard cell width, and guard cell arc length were measured on a single stomate basis using PI-stained, z-stack images of stomata from 3- to 4-week-old plants. Values are presented as mean ± SE (n ≥ 15 stomata per genotype per treatment from two independent experiments). Lowercase letters represent significantly different groups (P < 0.05, one-way ANOVA and Tukey test; ANOVA was performed within each treatment)*.

Considering the large amount of deformation that stomatal guard cells undergo during opening and closing, geometric nonlinear analysis was used. The guard cell wall was modeled to be a linear, orthotropic, elastic material. Because guard cell walls undergo repeated, reversible deformations, no plastic deformation was assumed. Therefore, sets of optimal elastic properties, which resulted in final stomatal geometries that matched the measured values of open stomata (Table 1), were sought by examining a full factorial combination of mechanical properties of orthotropic elasticity. The elastic mechanical properties of the guard cell wall were defined by three orthogonal axes representing longitudinal (polar, *E*_1_), circumferential (azimuthal, *E*_2_), and thickness (radial, *E*_3_) directions, as well as by shear moduli between two axes, including *G*_*12*_, *G*_*13*_, and *G*_*23*_, respectively (Supplemental Figures 3, 4). To describe stiffness in three directions relevant to the guard cell wall, the orientation of material properties was defined to reflect the arrangement of cellulose microfibrils in stomatal complexes (Supplemental Figure 4).

Poisson's ratio of the outer wall in onion epidermal fragments has been estimated to be approximately 1 from the movement of individual cellulose microfibrils (Zhang et al., 2017). However, Poisson's ratio for whole plant cell walls has yet to be experimentally determined. Here, Poisson's ratio of the guard cell wall was estimated using a cell wall network model (Yi and Puri, 2012) with simulated orthogonal deformation relative to the loading direction. Poisson's ratio determined for five network models of cell walls (Yi and Puri, 2012) is 0.003, which was assigned for all directions and was not varied in the process of finding optimal elastic properties, since in our FEMs, modulating Poisson's ratio between 0 and 0.49 had negligible effects on modeled stomatal geometries (*p* > 0.05, Mann-Whitney-Wilcox test).

In addition, thicknesses of guard cell walls were assigned based on their locations using thickness measurements of guard cell walls (Supplemental Table 1). Changes in wall thickness along the longitudinal axis of guard cells were deduced from images from Zhao and Sack (1999).

For full factorial analysis of cell wall properties, elastic properties were varied to be 200 kPa, 200 MPa, and 200 GPa, representing the widest range of possible cell wall stiffnesses. For example, 200 GPa represents a maximum stiffness value reported for crystalline cellulose based on density functional calculations (Nishiyama et al., 2008; Cintrón et al., 2011; Quesada Cabrera et al., 2011; Dri et al., 2013; Wu et al., 2013). On the opposite extreme, 200 kPa represents minimum reported stiffness values for plant cell assemblies under extension (Vanstreels et al., 2005; Zamil et al., 2014, 2015; Kim et al., 2015). Finally, (Wei and Lintilhac, 2007) reported moduli of cell walls to range from 213 MPa for young samples to 360 MPa for older samples. Therefore, the modulus of 200 MPa was chosen as a value in between the other two bounding values.

Based on these three levels of elastic modulus values, we created models of guard cell walls with full factorial combinations of Young's modulus in three orthogonal directions, namely E_1_ (polar), E_2_ (azimuthal), and E_3_ (radial), and three shear moduli, namely *G*_*12*_, *G*_*23*_, and *G*_*13*_. Inclusion of shear modulus accounts for the guard cell wall's change in shape that can significantly constrain its mechanical deformation. Based on pressure probe data for *Vicia faba* guard cells (Franks et al., 1998, 2001), a 5 MPa turgor increase was imposed to simulate opening of stomatal complex FEMs in 4 genotypes, with 10 models for each genotype and 729 different combinations of elastic moduli. The combinations of mechanical properties that resulted in open stomatal geometries that were closest to the observed stomatal geometries were further refined for each genotype using Broyden's method (Broyden, 1965) with respect to pore width (Figure 2A and Supplemental Movie 1).

We did not aggregate target guard cell geometries, because different stomatal geometries exhibited different sensitivity to different elastic moduli. This strategy differs from approaches used by recently published studies (Carter et al., 2017; Marom et al., 2017; Woolfenden et al., 2017) in which a single, simplified stomatal complex was modeled. In our approach, multiple imaging-derived FEMs were generated for each genotype, and an orthotropic material model was chosen to reflect the anisotropic nature of plant cell walls in all three orthogonal directions. Our approach inherently considers biological variability in the shape and size of stomatal complexes and, therefore, is free of potential confounding effects from simplifications of stomatal complex geometry. Typically, simulated stomatal geometries were within 10% of experimentally measured geometries.

### Statistical analysis

Statistical analyses of experimentally observed data were performed using the PAST statistics software package (Hammer et al., 2008). Statistical analyses of computational modeling results were performed using R (Ver. 3.3.2, R Core Team, 2016). Comparisons of measured and modeled stomatal geometries, including pore width, pore area, stomatal complex length, and guard cell width were conducted with Mann-Whitney-Wilcox tests. Effects of genotype on closed and open stomatal geometries were analyzed using ANOVA and Tukey tests. Graphics were produced with the ggplot2 package (Wickham, 2009) within the R environment.

## Results

### From 3D imaging to 3D modeling of stomata

To capture stomatal shape in three dimensions, we used propidium iodide (PI) to stain intact leaves of Col-0, *cesa3*^*je*5^, *xxt1 xxt2*, and *PGX1 OE* genotypes that had been treated with light or darkness to induce stomatal opening or closure, respectively. We then collected z-stack confocal images of stomata, which allowed us to generate XY, XZ, and YZ projections of single stomatal complexes (Figures 2A,B, Supplemental Movies 1, 2). Next, we traced along the midpoints of cell walls from the z-stack images of closed stomata of each genotype to extract landmarks (Figures 2C,D). Midpoints were traced from YZ projections and served as the scaffolds of our FEMs (Figure 2E). FE shell elements were defined with vertices representing the midpoint of the thickness of the guard cell wall. Because guard cell walls are differentially thickened (Zhao and Sack, 1999), we measured wall thickness at five different regions of a guard cell (Supplemental Figure 1 and Supplemental Table 1) and assigned these thickness values to the FE shell elements corresponding to each region. This approach enabled us to quantitatively investigate geometric changes during stomatal opening and to model interactions between guard cells and surrounding pavement cells without inserting idealizations or assumptions about the sizes and shapes of stomatal complexes (Franks et al., 1998, 2001).

To account for biological variability between stomata, ten stomatal complexes per genotype in the closed state were used to extract landmarks (Supplemental Figure 2). Using multiple stomatal complex FEMs increased the required computational resources but relieved a need for the introduction of mathematical averaging or variability calculations to account for the inherent variability of guard cell shape and size in real plants.

In our FEMs of open stomata (Figure 2E), stomatal pore geometries and guard cell geometries were calculated (Supplemental Table 2). Stomatal pore geometry includes pore width (aperture), pore length, and pore area (Figure 1). Guard cell geometry includes guard cell width and guard cell arc length (Figure 1). Measured geometries from 3D z-stack images of stomata in the open state (Figure 2A and Supplemental Movie 1) are listed in Table 1 and were used to validate FE modeling results (Supplemental Table 2).

### Effects of simplifying stomatal complex geometry on stomatal opening

**Figure 3:**
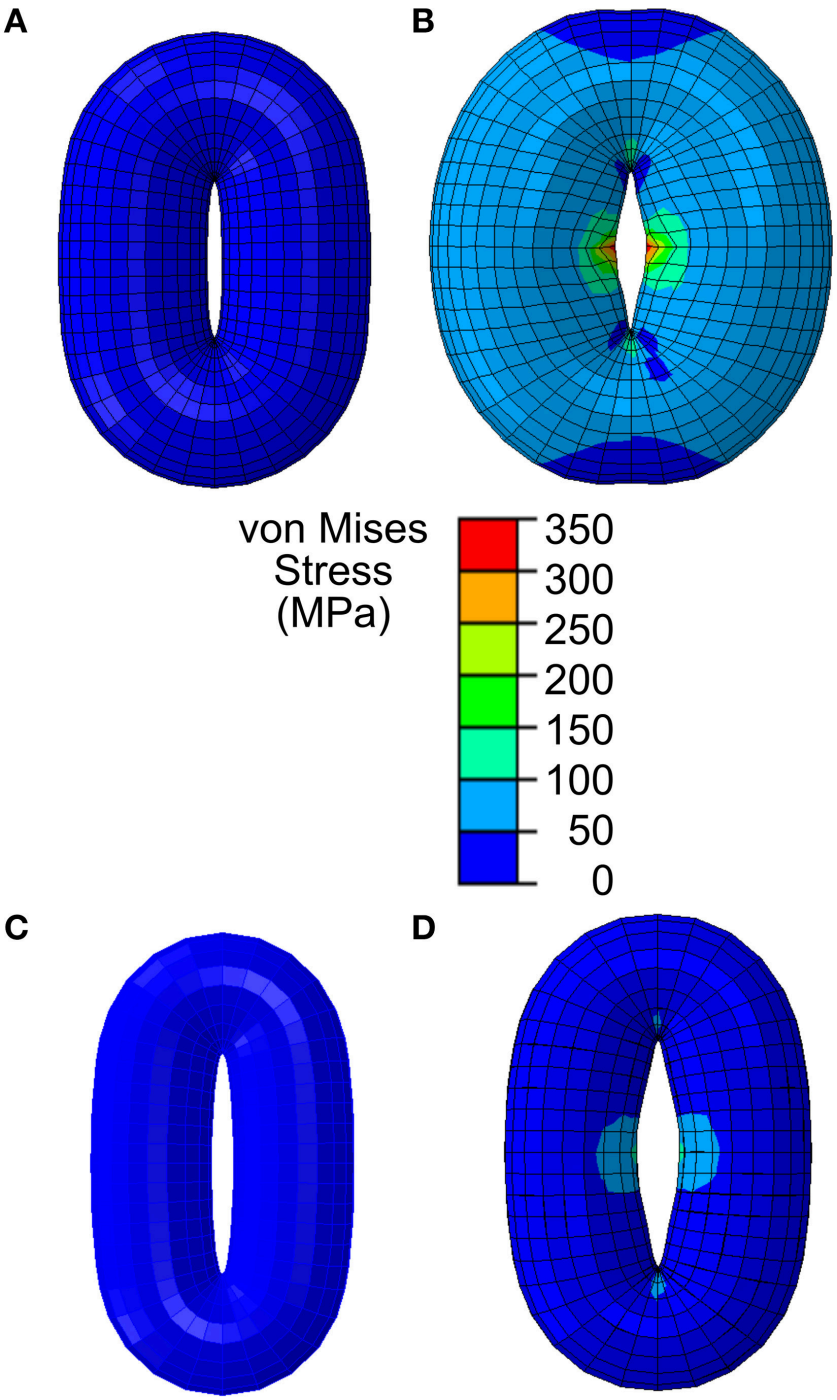
Stomatal opening simulation using a simplified guard cell model constructed as a torus using an elliptical stomatal pore profile when closed. **(A)** Stomatal guard cell model with a simplified geometric representation as a torus according to the reported Arabidopsis wild-type geometry in Woolfenden et al. (2017). **(B)** When stomatal opening is modeled in accordance with guard cell wall properties reported in Woolfenden et al. (2017), stomatal opening is slightly (10% based on the open pore width) underestimated. **(C)** Stomatal guard cell model with a simplified geometric representation as a torus according to the observed Col-0 (Table 1). **(D)** When this is modeled in accordance with guard cell wall properties reported in Woolfenden et al. (2017), stomatal opening is underestimated (40% based on the open pore width).

To examine how simplifying stomatal geometry affects the modeling of stomatal dynamics, we constructed a simplified model with following assumptions: (1) stomata open upon a 5 MPa turgor pressure increase, (2) guard cell wall thickness is 0.5 μm, (3) guard cell wall deformation is influenced by transverse shear deformation (thick shell model), (4) the guard cell wall is elastic, meaning that a stomatal complex recovers its original shape when guard cells are not turgid, and (5) there is no preemptively imposed symmetry, allowing for anisotropy in the guard cell wall in all directions. In addition, considering that the deformation of a stomatal complex is much larger than one percent of the original dimensions, the secondary effects of large deformations were considered (non-linear elasticity). In a simplified stomatal complex model, an ellipse and an elliptical torus (Woolfenden et al., 2017) were used to represent the stomatal pore and the guard cell pair, respectively (Figure 3).

Based on previous findings of the anisotropic behavior of stomatal guard cells (Cooke et al., 1976; Meckel et al., 2007; Woolfenden et al., 2017) and considering our stomatal measurements (Table 1), we also assumed that the guard cell wall is mechanically anisotropic. Specifically, considering the circumferential arrangement of cellulose microfibrils (Zhao and Sack, 1999), an “orthotropic isotropy” was assumed, which means that cell wall stiffness exhibits elastic symmetry with respect to three orthogonal planes. In our simplified stomatal complex models, the mechanical properties of *Arabidopsis* guard cell walls estimated by Woolfenden et al. (2017) were converted to values for orthotropic moduli. These values are listed in Table 2.

**Table 2:** Engineering elastic constants equivalent to Woolfenden et al. (2017)'s mechanical properties for wild-type *Arabidopsis* (All values are in MPa, 10^6^ N/m^2^).

**Genotype**	***E_1_***	***E_2_***	***E_3_***	***G_12_***	***G_13_***	***G_23_***
Col-0	99	601	601	33	33	33

*Poisson's ratio (ν) was assigned to be 0.2 to ensure the positive strain energy and determinant of the compliance matrix*.

When opening of a geometrically simplified stomatal complex was simulated using the initial dimensions reported in Woolfenden et al. (2017) as shown in Figure 3A, stomatal pore width increased from 0.9 to 1.8 μm (Figure 3B). Considering that simple linear elasticity was used to model cell wall mechanics and that the converted modulus values do not involve hyperelasticity theory, this 10% underestimation is remarkably close to the results of Woolfenden et al. (2017). However, when a guard cell pair was modeled with simplified geometry (ellipse plus elliptical torus) but also with initial dimensions taken from actual stomatal complexes (Table 1) as shown in Figure 3C, stomatal pore width increased from 1.2 to 2.3 μm (Figure 3D). This is a 41% underestimation when compared to observed stomatal geometries (Table 1).

### Effects of pavement cell constraints on stomatal opening

Another important aspect of a stomatal complex is its interaction with pavement cells. To isolate the effects of pavement cell constraints on stomatal dynamics, stomatal opening was modeled with realistic models of stomatal complexes of *Arabidopsis* Col-0 that were based on measured stomatal geometries (Figure 2). The mechanical properties of the guard cell walls were consistent with those in the simplified stomatal complex models (Figure 3).

Stomatal opening was first modeled without any constraints around the stomatal complex. Next, stomatal opening was modeled using observed stomatal complex widths and lengths under closed and open conditions (Table 1) as constraints.

**Figure 4:**
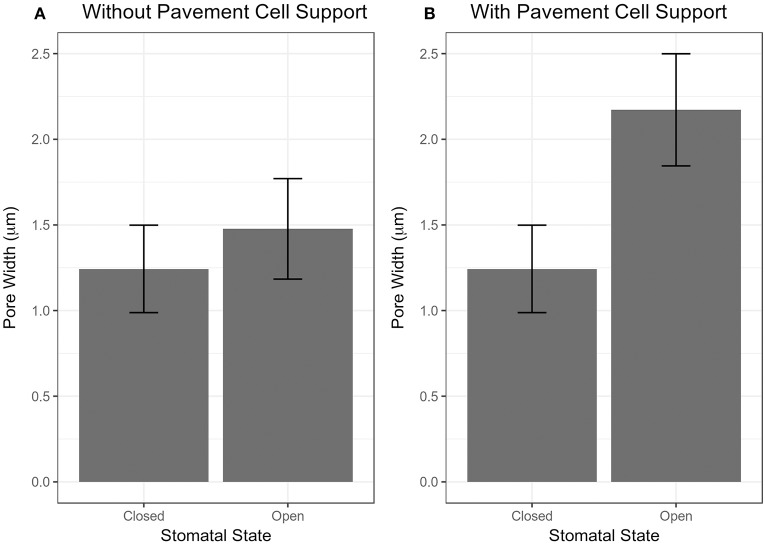
Results of stomatal opening simulation with realistic stomatal complex models of *Arabidopsis* Col-0 without **(A)** and with **(B)** mechanical support from neighboring pavement cells. Simulated opening of traced stomatal FE models was performed using wall mechanical properties equivalent to Woolfenden et al. (2017) (Table 2).

The importance of pavement cell constraints on a stomatal complex is illustrated in Figure 4. Figure 4A shows an underestimation in stomatal opening when no constraint was included, but when observed stomatal complex widths and lengths were incorporated as size constraints on the stomatal complex due to mechanical constraints imposed by neighboring pavement cells, stomatal pore width increased significantly upon simulated pressurization of 5 MPa (Figure 4B; *p* < 0.05, Mann-Whitney-Wilcox test).

### Effects of modified wall composition on stomatal geometry

**Figure 5:**
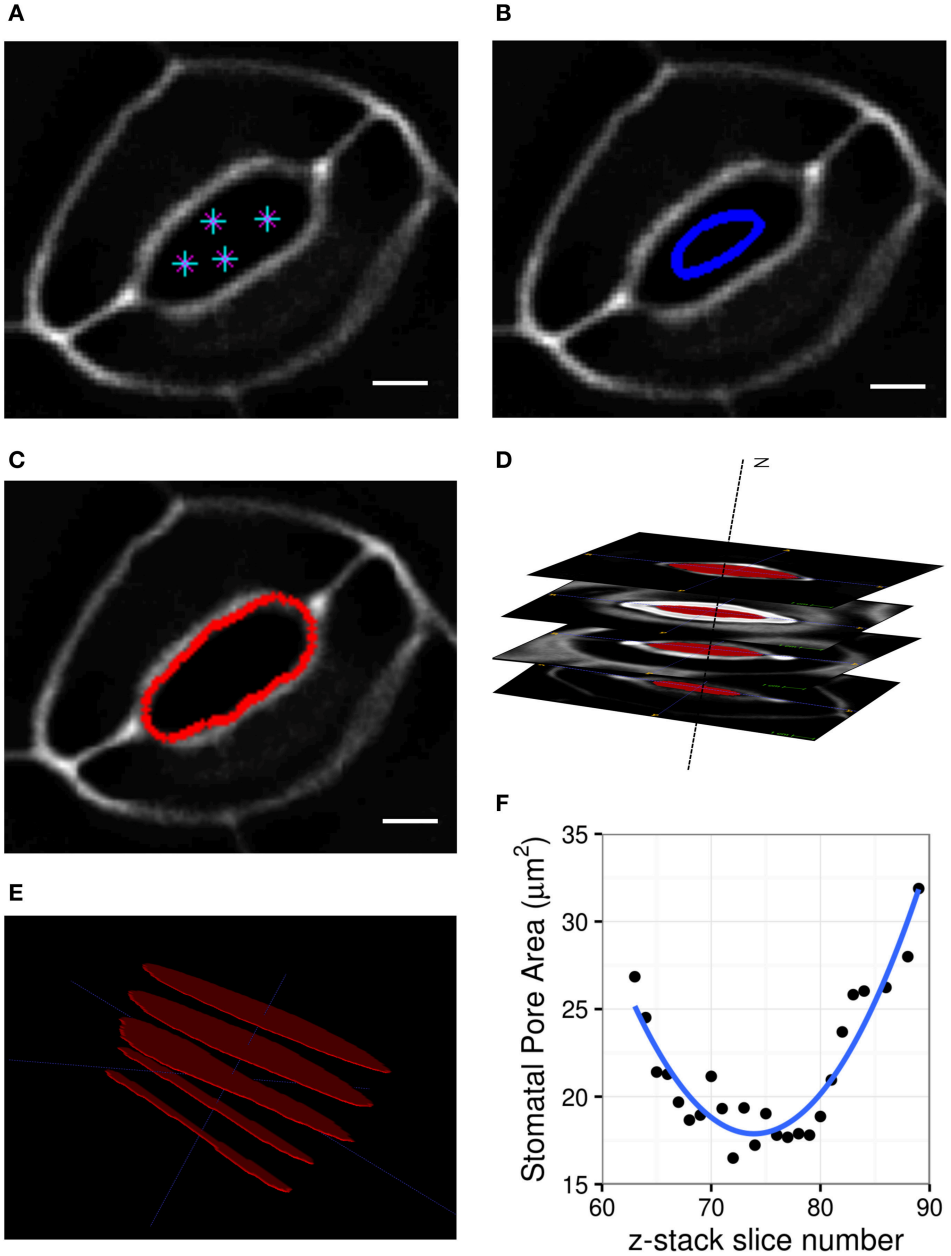
Procedure of stomatal pore area measurement. **(A)** A few points are input by the user on a single slice of the z-stack. **(B)** A curve is initialized from these points. **(C)** The curve expands and evolves according to edges in the image and fits the pore opening. **(D)** Cross-sectional view of detected pore areas after segmentation through a z-stack. **(E)** Pixel counts within the extracted pore areas are calculated, and the smallest one is converted to pore area in μm^2^. **(F)** Stomatal pore areas of z-stack slices are fitted to a robust quadratic equation to find the respective minimum value representing the pore area of a given stomatal complex. Scale bars represent 5 μm.

**Figure 6:**
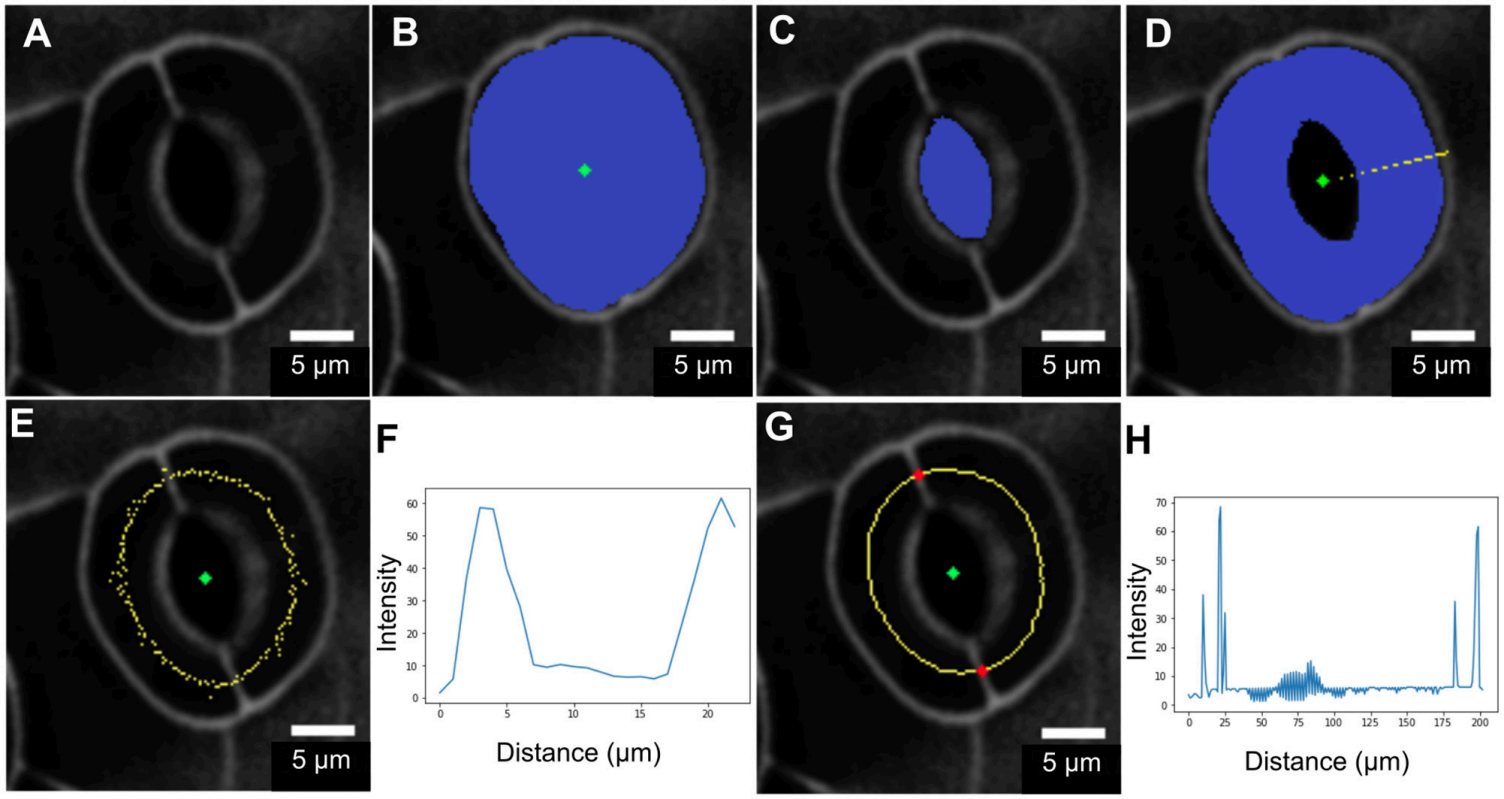
Procedure for measuring guard cell arc length. **(A)** Original image. **(B)** Segmentation of the stomatal complex via active contours. **(C)** Pore area segmentation via active contours. **(D)** Cell pair area extraction through a binary morphological operation (XOR). **(E,F)** Non-smooth midline (yellow) calculated between outer cell boundaries and pore boundaries. Intensity profile analysis is in the radial direction. **(G,H)** Mid-points after smoothing and edge-filling. Junction point detection (red) via intensity profile analysis along the midline.

Geometric parameters of stomata, including pore width, pore length, pore aspect ratio (width/length), pore area, stomatal complex length, stomatal complex width, guard cell arc length, guard cell width, and guard cell junction area (Figure 1 and Table 1), were determined computationally or manually from experimentally acquired images of PI-stained stomatal complexes, depending on the accuracy of computational segmentation as benchmarked against subsets of manual measurements. Three-dimensional features, such as the irregular border of a stomatal pore, were taken into account in our measurements, since pore shape did not conform to idealized geometries (e.g., an ellipse or a symmetric lens). Computational image analysis enabled bias-free measurements of geometry. Both our computational and manual image analyses searched for the narrowest stomatal pore width (Figure 5), and the largest dimensions for stomatal complex length, stomatal complex width, and guard cell arc length (Figure 6) throughout the 3D space occupied by the actual stomatal complex.

When comparing measurements of stomatal pore geometry across genotypes (Table 1), the trends of pore width changes using the 3D confocal image datasets were consistent with what we have previously observed in 2D brightfield image datasets (Rui and Anderson, 2016): *cesa3*^*je*5^ stomata had larger pore widths, whereas *xxt1 xxt2* stomata exhibited smaller pore widths, than wild type controls in the open or closed state (Table 1) (Rui and Anderson, 2016). *PGX1 OE* stomata showed the greatest increase in pore width, pore aspect ratio, and pore area from the closed state to the open state upon experimentally induced opening, whereas *xxt1 xxt2* stomata showed the smallest increases in these values among the four genotypes (Table 1).

The function of stomata in regulating gas exchange by controlling stomatal conductance (von Caemmerer and Farquhar, 1981) is assumed to be directly related to pore area (Franks and Farquhar, 2001; Franks et al., 2009). Therefore, we performed correlation analyses between pore width and pore area in either closed or open state and found that these two parameters fit in a linear relationship for all four genotypes, regardless of the functional state of the stomata (Supplemental Figure 5).

When comparing measurements across genotypes (Table 1), stomatal complex length showed a significant increase from the closed to the open state in *xxt1 xxt2* mutants, whereas the other genotypes displayed small changes in stomatal complex length between the two functional states. The pattern of changes in stomatal complex width was consistent with the trend of pore width changes, i.e., *PGX1 OE* plants showed the most increase, whereas *xxt1 xxt2* mutants had the least increase in stomatal complex width from the closed state to the open state among all four genotypes (Table 1).

When comparing guard cell junction areas, which were analyzed manually from YZ projections of 3D confocal images (Figure 1), we found a significant reduction in guard cell junction area from the closed state to the open state in Col-0 controls, but not in genotypes with altered wall composition (Table 1).

**Figure 7:**
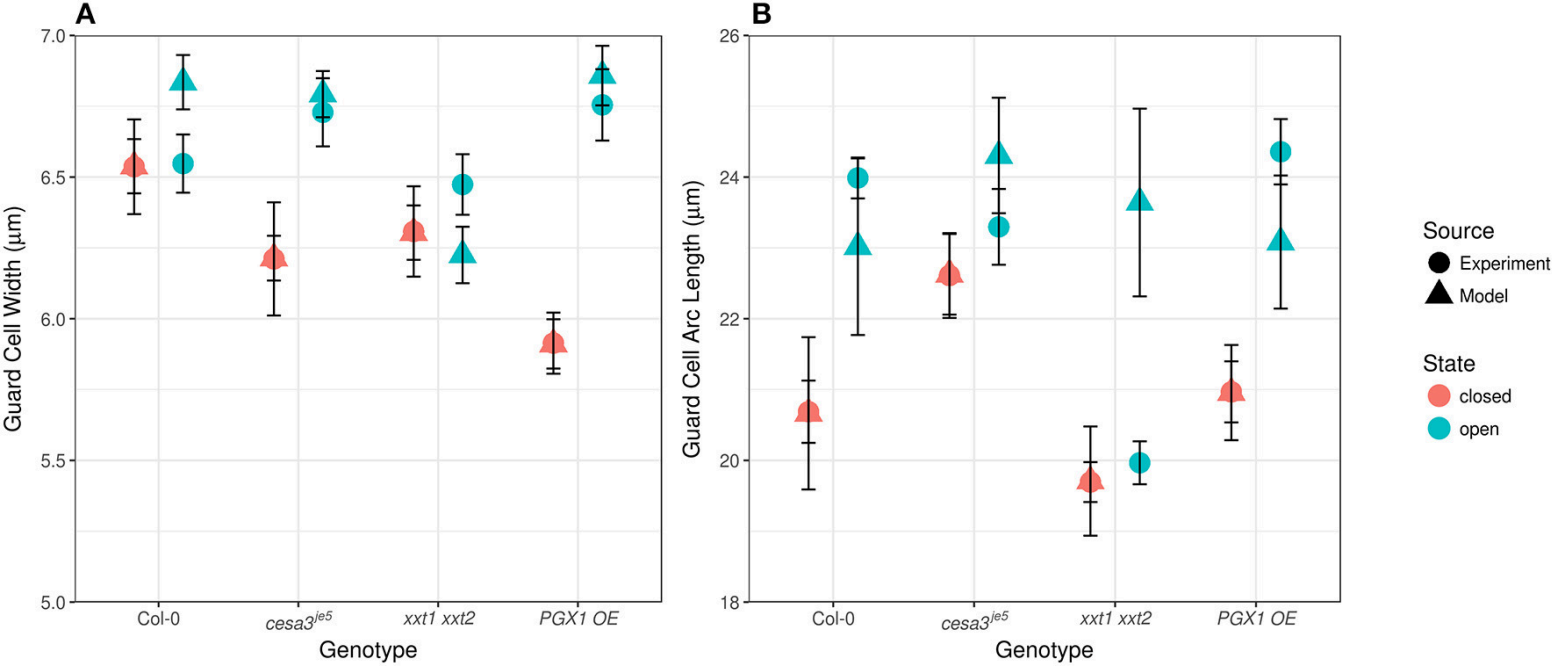
Experimentally observed and computationally predicted guard cell widths and lengths of Col-0, *cesa3*^*je*5^, *xxt1 xxt2*, and *PGX1 OE* stomata. **(A)** Error bars represent standard error. Guard cell width (*n* > 15) did not change upon opening for Col-0, *cesa3*^*je*5^, and *xxt1 xxt2* genotypes (*p* > 0.05 for all three genotypes, Mann-Whitney-Wilcox test), whereas *PGX1 OE* showed radial expansion upon opening (*p* < 0.001, Mann-Whitney-Wilcox test). FE simulation (*n* = 10 for each genotype) predicted non-significant radial expansion for Col-0 and *xxt1 xxt2* (*p* > 0.05 for both genotypes, Mann-Whitney-Wilcox test) but significant radial expansion for *cesa3*^*je*5^ (*p* = 0.052, Mann-Whitney-Wilcox test) and *PGX1 OE* (*p* < 0.001, Mann-Whitney-Wilcox test). **(B)** Guard cell arc length (*n* > 11) changed significantly only for Col-0 and *PGX1 OE* upon opening (*p* < 0.001, Mann-Whitney-Wilcox test), whereas guard cell arc length did not change significantly for *cesa3*^*je*5^ and *xxt1 xxt2* stomata (*p* > 0.05 for all three genotypes, Mann-Whitney-Wilcox test). However, guard cell arc length in the open state from FE model simulation (*n* = 10 for each genotype) did not differ significantly from that in the closed state (*p* > 0.05, Mann-Whitney-Wilcox test) except for *xxt1 xxt2* (*p* = 0.03, Mann-Whitney-Wilcox test).

Comparing measured values for guard cell geometry that were not used in constructing our FEMs (Table 1 and Figure 7) to modeled values highlights additional mechanical forces in stomatal dynamics. For example, we did not observe significant changes in guard cell width between closed and open stomata in Col, *cesa3*^*je*5^, or *xxt1 xxt2* from our 3D confocal image dataset, a finding consistent with 2D brightfield imaging (Rui and Anderson, 2016), but guard cell width did increase significantly upon opening in *PGX1 OE* stomata (Figure 7A; *p* < 0.001, Mann-Whitney-Wilcox test). Similarly, FEM results showed non-significant changes in guard cell width for Col, *cesa3*^*je*5^, or *xxt1 xxt2* (*p* > 0.05, Mann-Whitney-Wilcox test), but showed a significant increase for *PGX1 OE* (*p* < 0.001, Mann-Whitney-Wilcox test). However, for Col, FEM results significantly overestimated changes in guard cell width compared to experimental measurements (*p* = 0.01, Mann-Whitney-Wilcox test).

Compared to guard cell width, guard cell arc length, as measured experimentally, increased significantly from the closed to the open state only for Col-0 and *PGX1 OE* stomata (Figure 7B). This observation is consistent with previous findings that guard cells elongate to open the stomatal pore (Meckel et al., 2007; Rui and Anderson, 2016). Although FEM results replicated experimental observations for Col, *cesa3*^*je*5^, and *PGX1 OE* (*p* > 0.05, Mann-Whitney-Wilcox test), FEM overestimated guard cell arc length in the open state in *xxt1 xxt2* mutants compared to experimental measurements (*p* < 0.001, Mann-Whitney-Wilcox test). The fact that guard cell arc length did not increase significantly in experimental measurements of *xxt1 xxt2* or *cesa3*^*je*5^ stomata, and that discrepancies between measured and modeled arc length existed for *xxt1 xxt2*, suggests that guard cell elongation might not be the only driving mechanism for stomatal opening, since stomata opened to at least some degree in all genotypes.

### Modeled mechanical properties of guard cell walls

Ranges of mechanical properties resulting in open stomatal geometry that matched experimental observations (*p* > 0.05, Mann-Whitney-Wilcox test) are listed in Table 3. These ranges of mechanical properties were identified for stomatal complexes of each genotype when we removed physically impossible sets (i.e., violating symmetry required by orthotropic elasticity) and biomechanically impossible sets (i.e., stiffness higher than the known maximum stiffness of cellulose) (Cintrón et al., 2011; Quesada Cabrera et al., 2011). The estimated mechanical properties of guard cell walls (Table 2) were within the ranges of experimental measurements of wall mechanical properties (Vanstreels et al., 2005; Peaucelle et al., 2011; Zamil et al., 2013, 2014, 2015), including those generated from nanoindentation experiments such as atomic force microscopy (AFM) (Milani et al., 2011; Peaucelle et al., 2011; Forouzesh et al., 2013; Carter et al., 2017). Furthermore, for each genotype, a single combination of mechanical properties that result in open stomatal geometry to be the closest to experimental observations was identified (values in parentheses of Table 3). The trends of the listed ranges of mechanical properties are consistent with the optimal values. Therefore, for simplicity, optimal values are used in the following sections.

**Table 3:** Ranges of elastic moduli values including Young's moduli (*E*_*1*_*, E*_*2*_, *and E*_*3*_*)* and shear moduli (*G*_*12*_, *G*_*13*_, and *G*_*23*_) of FE guard cell models that reproduces statistically indistinguishable (*p* > 0.05 Mann-Whitney-Wilcox test) open stomatal pore width (All values are in MPa, 10^6^ N/m^2^).

**Genotype**	***E_*1*_***	***E_*2*_***	***E_*3*_***	***G_*12*_***	***G_*13*_***	***G_*23*_***
Col-0	0.01–1 (0.5)	0.2–6 (2)	0.2–6 (2)	5–50 (50)	0.1–50 (10)	0.1–50 (10)
*cesa3^*je*5^*	0.1–10 (5)	0.2–25 (25)	0.2–25 (25)	0.1–10 (10)	0.1–10 (10)	0.1–10 (10)
*xxt1 xxt2*	0.0025–0.25 (0.0025)	0.0002–0.2 (0.0002)	0.0002–0.2 (0.0002)	0.25–1 (0.25)	0.25–1 (0.25)	0.5–2 (0.5)
*PGX1 OE*	0.1–1 (0.1)	1–10 (10)	1–10 (10)	1.5–10 (10)	1.5–10 (10)	50–750 (750)

*Values in parentheses are values of elastic moduli that resulted in open stomatal geometries that were closest to experimental observations*.

In all genotypes except *xxt1 xxt2*, longitudinal modulus (*E*_*1*_*, polar*) was smaller than moduli in other directions (*E*_2_*, azimuthal* and *E*_3_*, radial*) (Table 3). The anisotropy ratios of *E*_*1*_ to both *E*_2_ and *E*_3_ were 1:4 and 1:5 for Col-0 and *cesa3*^*je*5^, respectively. *PGX1 OE* required a much higher anisotropy ratio of *E*_1_ to both *E*_2_ and *E*_3_ (1:100) to achieve the observed stomatal opening. For Col-0, it is notable that shear moduli (*G*_12_, *G*_13_, and *G*_23_) were higher than Young's moduli (*E*_1_*, E*_2_, and *E*_3_), which explains the limited amount of circumferential expansion of guard cells during stomatal opening. In addition, a higher *G*_12_ than *G*_13_ and *G*_23_ suggests that the interaction between cellulose and matrix polymers may form preferentially along the longitudinal axes of CMFs, which is the circumferential direction in a guard cell, resulting in similar molecular structures and mechanical properties in the shear planes involving the radial (thickness) direction.

Altered mechanical properties in mutants provided insights into the relationship between their modified wall structures and mechanical behaviors. For *cesa3*^*je*5^, which has a reduced amount of cellulose (Rui and Anderson, 2016), Young's moduli counterintuitively were predicted to increase in all three directions (*E*_1_, *E*_2_, and *E*_3_) (Table 3). Modeled shear moduli were lower in the longitudinal and circumferential directions than in the radial direction in *cesa3*^*je*5^ stomata.

For FEMs of the *xxt1 xxt2* genotype, which lacks xyloglucan (Cavalier et al., 2008), Young's moduli were predicted to be substantially lower than Col-0 moduli in the longitudinal, circumferential, and radial directions (Table 3). In addition, *E*_1_ was larger than *E*_2_ and *E*_3_, a trend of anisotropic stiffness that is contrary to other genotypes. Combinations of these changes and additional constraints of neighboring cells might limit stomatal opening in *xxt1 xxt2*, as experimentally observed. In addition, the lower stiffness in *xxt1 xxt2* guard cell walls suggests that a lack of xyloglucan significantly hampers the load-bearing mechanism of guard cell walls in normal deformations, which corroborates Park and Cosgrove (2015) finding. However, the smaller decreases in shear moduli than Young's moduli in *xxt1 xxt2* suggest that the mechanism responsible for shear load bearing might involve xyloglucan and that it could be partially compensated for by other cell wall constituents, such as pectic polysaccharides, in the absence of xyloglucan.

For *PGX1 OE*, which has pectic polysaccharides with smaller molecular weight (Xiao et al., 2014), modeled changes in Young's modulus in the FEMs manifested differently in each direction. Compared to their counterparts in Col-0 controls, *E*_1_ was lower, but *E*_2_ and *E*_3_ were higher in *PGX1 OE* guard cells. A lower *E*_1_ value suggested that the decreased molecular weight of pectins might make it easier to stretch the guard cell in the longitudinal direction. Increases in *E*_2_ and *E*_3_ suggested a crucial role for pectins in the guard cell wall to achieve an appropriate level of stiffness in circumferential and radial directions (Rui et al., 2017). This suggests that a reduction in pectin molecular weight interferes with the load-bearing capability of the wall in the longitudinal direction, while the wall stiffens in the circumferential and radial directions. Increased pore opening in *PGX1 OE* stomata seemed to relate inversely to the longitudinal stiffness of the guard cell wall, as might be intuitively expected.

In *PGX1 OE* FEMs, we also observed a much higher *G*_23_ than that in Col-0 controls. This increase in *G*_23_ suggested that in wild type stomata, large, flexible pectin molecules might serve as elastic buffers between layers of cellulose. In addition, the increased connectivity of networks of smaller pectic polysaccharides might inhibit wall deformation in the circumferential direction while moderately weakening mechanical stiffness in the longitudinal direction.

### Effects of wall composition on the dynamic geometry of stomatal complexes

Whereas Col-0 stomata opened from 1.2 to 3.9 μm in response to light treatment, *cesa3*^*je*5^ stomata opened from 2.5 to 4.7 μm (Table 1). Although *cesa3*^*je*5^ stomatal pore widths were larger than Col-0 pore widths in both states (Table 1), the ratio of open to closed pore widths was smaller in *cesa3*^*je*5^ (2:1) than that in Col-0 (3:1). This was reflected as an increase in the *E*_1_, *E*_2_, and *E*_3_ values for FEMs of *cesa3*^*je*5^ guard cell walls (Table 3). Smaller shear moduli of *cesa3*^*je*5^ stomata in longitudinal and circumferential directions suggest a loss of resistance in the circumferential direction upon deformation in the longitudinal direction. Overall, considering that *cesa3*^*je*5^ guard cells were predicted to have stiffer cell walls, increased pore opening of *cesa3*^*je*5^ stomata was attributable more to altered initial stomatal geometry in comparison to Col-0 guard cells, rather than to reductions in wall stiffness in this cellulose-deficient mutant. Stomatal pore width was smaller in both open and closed *xxt1 xxt2* stomata, and the ratio of open:closed stomatal pore width was larger than that of Col-0 stomata. Reflecting the observed stomatal opening and other geometric changes, the modeled stiffness of *xxt1 xxt2* guard cell walls was substantially smaller than the stiffness of Col-0 walls (Table 3). The *PGX1 OE* open:closed pore width ratio (6:1) was the largest among all genotypes, which was reflected in reductions in *E*_1_ and *G*_12_ (Table 3).

Counterintuitively, *E*_2_ and *E*_3_ did not directly correlate with changes in guard cell width during stomatal opening. For Col-0, guard cell width did not change upon opening (*p* = 0.85, Mann-Whitney-Wilcox test). With lower *E*_2_ and *E*_3_ values, as in the case of *xxt1 xxt2*, guard cell width also remained the same upon opening (*p* = 0.68, Mann-Whitney-Wilcox test). However, when *E*_2_ and *E*_3_ were higher, as in case of *cesa3*^*je*5^ and *PGX1 OE* stomata, guard cell width still remained the same upon opening for *cesa3*^*je*5^ (*p* = 0.051, Mann-Whitney-Wilcox test), but increased for *PGX1 OE* (*p* = 0.00002, Mann-Whitney-Wilcox test), indicating radial expansion of guard cells in the latter genotype. The radial expansion of guard cells might reduce pore opening, similarly to the elliptical shape of guard cell cross sections proposed by (Cooke et al., 1976), and thus be detrimental to achieving a maximal stomatal pore area. It seems that the ratio between Young's moduli (*E*_1_, *E*_2_, and *E*_3_) and shear moduli involving the longitudinal direction (*G*_12_ and *G*_13_) are more intimately related to guard cell expansion during stomatal opening.

### Stress and strain distribution in guard cells with open stomata

**Figure 8:**
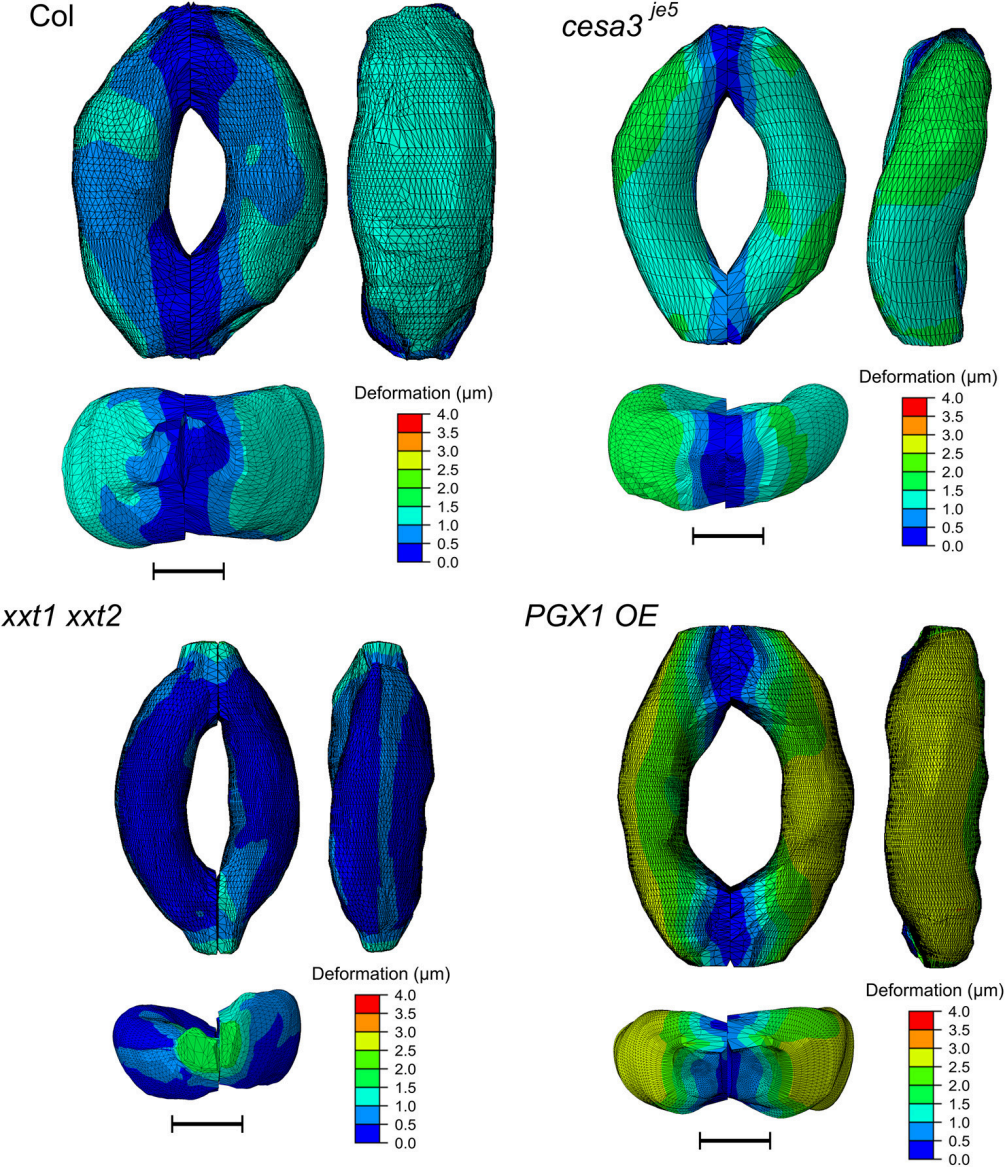
Typical results of FE modeling of stomatal guard cells of Col-0, *cesa3*^*je*5^, *xxt1 xxt2*, and *PGX1 OE* genotypes, showing distributions of deformation of guard cell walls upon simulated opening. FE models shown here has been processed with a Laplacian smoothing (Vollmer et al., 1999). Results of stomatal opening using original and smoothed FE models are identical. Overall, deformation is distributed throughout the guard cells, with less deformation at junction regions. Scale bars represent 5 μm.

Figure 8 shows typical results of modeled deformations of Col-0, *cesa3*^*je*5^, *xxt1 xxt2*, and *PGX1 OE* stomata, respectively, upon 5 MPa pressurization. In Col-0 FEMs, the amount of deformation was distributed evenly. For *cesa3*^*je*5^ and *PGX1 OE*, wall deformation tended to be higher near the cell midsection, and junction areas showed smaller deformation, which is consistent with a limited change in stomatal complex length compared to a significant change in stomatal complex width (Rui and Anderson, 2016), and is consistent with polar stiffening during stomatal opening (Carter et al., 2017). On the other hand, *xxt1 xxt2* FEMs show larger deformation near cell-cell junctions.

**Figure 9:**
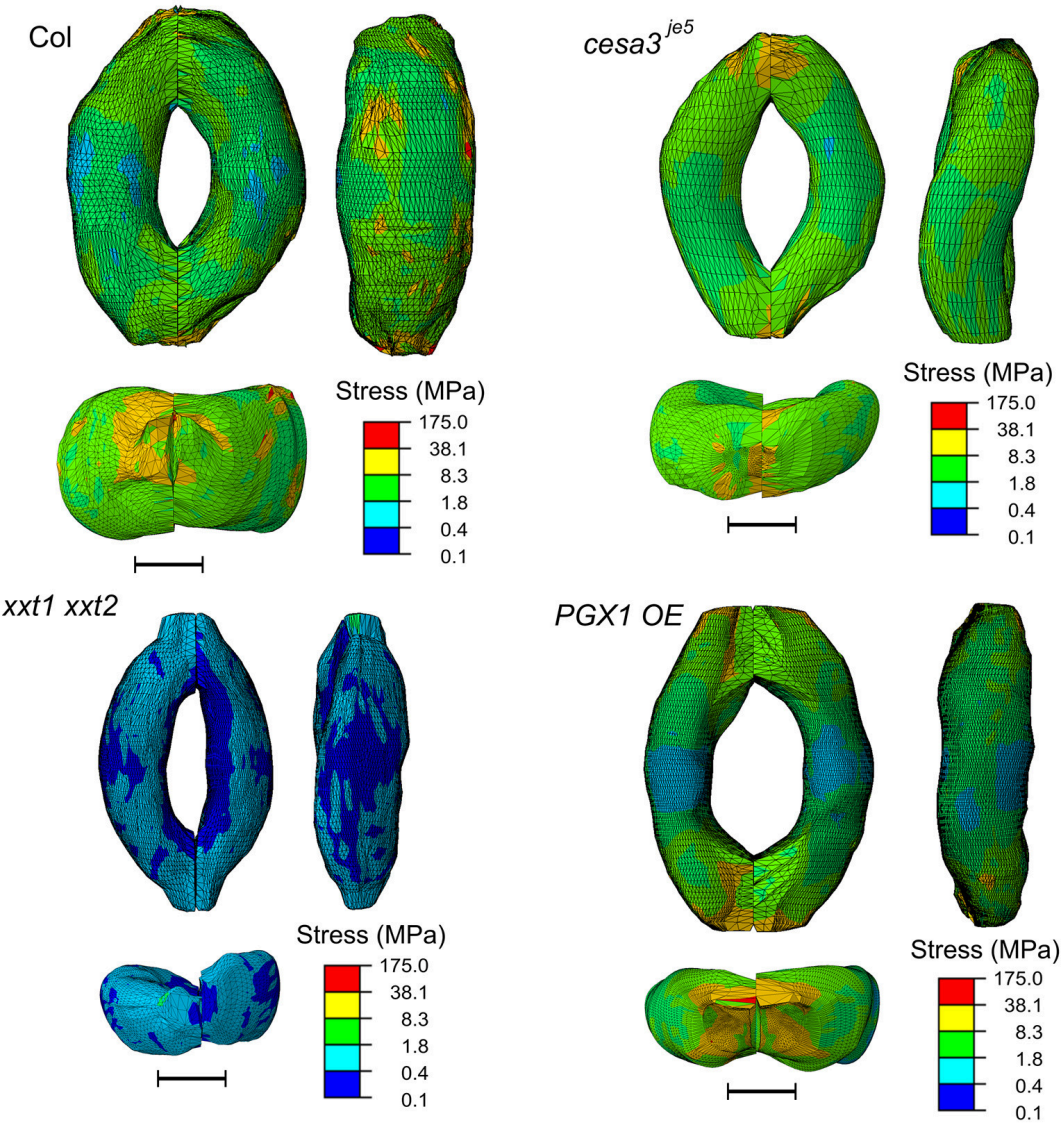
Typical stress distributions in FE models of Col-0, *cesa3*^*je*5^, *xxt1 xxt2*, and *PGX1 OE* guard cells upon simulated opening. FE models shown here has been processed with a Laplacian smoothing (Vollmer et al., 1999). Results of stomatal opening using original and smoothed FE models are identical. Overall, stress concentration is observed near guard cell junctions and corners. Scale bars represent 5 μm.

The concentration of deformations in specific regions might be predicted to hamper proper stomatal function after many rounds of stomatal movement, since these regions of the guard cell walls might be more subject to mechanical stress. However, the stress level of guard cell walls did not exhibit a similar spatial distribution (Figure 9). Noticeable stress concentration was observed near junctions and periclinal ridge regions. Dorsal or ventral regions did not show specific stress concentration, which might be advantageous for maintaining stomatal function across many rounds of opening and closure.

In our FEMs, additional loadings on junction and dorsal regions from neighboring cells were calculated in response to the imposed constraints on stomatal complex length and width determined by stomatal geometry measurements (Table 1). Loadings from neighboring cells on the dorsal regions of guard cells were similar for Col-0, *cesa3*^*je*5^, and *PGX1 OE*, whereas *xxt1 xxt2* FEMs required a much lower degree of loading (Table 4). Notably, constraints at the junction area needed to be much higher for Col-0 than for other genotypes, in which additional forces on junction area essentially vanished and it appeared that guard cells in these mutant genotypes lost mechanical support from neighboring cells (Table 4).

**Table 4:** Predicted additional loadings on stomatal guard cells at the dorsal side from neighboring cells and at junction area.

**Genotype**	**Loading on stomatal junction area (MPa)**	**Loading on stomatal dorsal area (MPa)**
Col-0	1.4	0.1
*cesa3^*je*5^*	−0.4	0.2
*xxt1 xxt2*	−0.3	0.1
*PGX1 OE*	−0.02	0.0001

## Discussion

### Implications of simplifying the geometry of stomatal complexes

A stomatal complex is a structure comprising a pair of guard cells that interact with neighboring subsidiary or pavement cells. Mechanically, a “structure” refers to a system of connected parts supporting imposed loads (Hibbeler, 2011). The following aspects influence the structure-load relationships that drive stomatal dynamics: (1) turgor pressure, (2) the shape and size of each guard cell, (3) guard cell wall thickness, (4) the geometry and configuration of connections between sister guard cells, (5) mechanical properties of the guard cell wall, and (6) support/constraint from neighboring cells. In modeling the opening and closing behavior of a stomatal complex, some of these aspects can be simplified. However, it is imperative to account for the implications of the assumptions that accompany each simplification in order to accurately interpret modeling results. Considering a stomatal complex as a mechanical structure, when the load imposed by turgor pressure, material model, modulus values, and thickness of the guard cell wall are set, there are two remaining important aspects to consider, namely connections between paired guard cells and interactions with neighboring cells. Here, we discuss implications from those aspects of simplified models of stomatal complexes and the benefits of using realistic models of stomatal complexes to overcome key limitations of the simplified models.

#### Guard cell geometry

In previous studies that model stomatal complexes, the overall shape of a guard cell pair or a stomatal pore has been simplified to be elliptic (DeMichele and Sharpe, 1973, 1974; Shoemaker and Srivastava, 1973; Cooke et al., 1976; Sharpe and Wu, 1978; Carter et al., 2017; Marom et al., 2017; Woolfenden et al., 2017). However, we observed significant asymmetry in many of the imaging-based FEMs we constructed, both at the subcellular scale, as evidenced by surface “bumpiness,” and at the cellular scale, with guard cells bulging slightly on the opposite ends as their sister cells (Figure 8, Supplemental Figure 2). These observations call into question the assumption that stomatal complexes are perfectly symmetrical, both longitudinally and transversely, when developing analytical and numerical models to represent stomatal dynamics (Cooke et al., 1976; Rui et al., 2016; Marom et al., 2017; Woolfenden et al., 2017).

Furthermore, assuming a simplified elliptical torus is a robust representation of a guard cell pair in a stomatal complex, simulation results of stomatal opening would be consistent under the same biomechanical conditions even when the geometry of a stomatal complex varies. From the comparison of two different stomatal complex geometries (Figure 3), it is clear that stomatal opening is sensitive to the overall guard cell shape.

Unfortunately, it is not straightforward to discern which geometric feature of a stomatal complex affects stomatal opening. For example, the shape of an open stomatal pore, which may be more relevant in stomatal functionality than pore width, is highly sensitive to the closed stomatal pore shape and the dorsal wall curvature of a guard cell. At the same time, stomatal junction length significantly affects stomatal pore enlargement in terms of the overall shape, maximum pore width, and pore area. Therefore, a simplified stomatal complex model aggregates such effects of simplification without a good way to validate their physical implications. By contrast, realistic stomatal complex models do not introduce assumptions about stomatal geometry, but instead reflect biological variations in stomatal geometry, including asymmetry between paired guard cells and any irregularity in stomatal pore shapes, thus avoiding any confounding effects of a simplified stomatal geometry.

#### Interactions with neighboring cells

*A* stomatal complex is connected to neighboring pavement cells. While a difference in turgor pressure between guard cells and pavement cells has not been quantitatively substantiated, it is clear that guard cells are supported by neighboring cells during stomatal opening and closing. Cooke et al. (1976) modeled this interaction as a component of boundary conditions. However, in recent modeling studies (Carter et al., 2017; Marom et al., 2017; Woolfenden et al., 2017), these interactions are not explicitly included.

As observed in Figure 4, pavement cell constraints on a stomatal complex have a significant effect on stomatal opening. The influence and importance of mechanical interaction between stomatal guard cells and pavement cells were originally investigated by DeMichele and Sharpe (1973), although the direction of mechanical advantage between guard cells and subsidiary cells are not always consistent.

In conclusion, considering the existing complexity of mechanically modeling the guard cell wall and the number of geometric features of a stomatal complex, it is impractical to investigate and optimize the effect of each simplified aspect in isolation as part of a multifactorial exercise in model building. This is especially true when investigating stomatal complexes in different genotypes as their size and shape change simultaneously. Therefore, a clear advantage of using a realistic stomatal guard cell model traced from 3D microscopic images is that this approach constrains the geometry of the stomatal complex, simplifying the parameter space of the model, and makes this geometry realistic.

### The anisotropic mechanical properties of guard cell walls reveal how major polysaccharides contribute to guard cell mechanics

Our calculated Young's moduli and shear moduli highlight the highly anisotropic mechanical properties of guard cell walls. Higher stiffness values in circumferential or azimuthal (*E*_2_) and radial (*E*_3_) directions than in the longitudinal or polar direction (*E*_1_) corroborate previous results (Yi and Puri, 2012, 2014). In addition, Young's moduli ranging from 2 to 25 MPa in the circumferential (*E*_2_) and radial (*E*_3_) directions and shear moduli of up to 750 MPa in the longitudinal plane (*G*_23_) are much higher than stiffness values expected from non-covalent interactions, e.g., between cellulose and xyloglucan (Hayashi, 1989; McQueen-Mason and Cosgrove, 1994), cellulose and pectins (Zykwinska et al., 2005), or xyloglucan and pectins (Rizk et al., 2000; Brett et al., 2005; Cumming et al., 2005; Popper and Fry, 2008). These results suggest that load bearing by cellulose in the circumferential and radial directions in guard cell walls is a possible origin of the higher stiffness we modeled in the circumferential (*E*_2_) and radial (*E*_3_) directions. In the circumferential direction (E_2_), an applied force is thought to be borne by the cellulose backbone. In the radial direction (E_3_), an applied force is more likely to be borne by the compressive or tensile resistance of cellulose rather than the cell wall matrix.

Given the apparent dominance of cellulose in determining the anisotropic mechanical properties of the wall, the increased longitudinal stiffness (*E*_1_) we modeled in *cesa3*^*je*5^ is counterintuitive. The deficiency of cellulose in guard cell walls results in overall guard cell enlargement, but not in enhanced radial expansibility (Rui and Anderson, 2016). Reflecting this result, in our FEMs of cellulose-deficient guard cell walls, stiffness increased greatly in the longitudinal direction, whereas it increased much less in the circumferential direction. However, shear modulus values, which constrain the radial expansion of guard cells, did not change as much (Table 2). These results suggest a direct correlation between cellulose content and wall stiffness in a direction perpendicular to CMF orientation (*E*_1_), which might be due to cellulose bundling and/or crosslinking by matrix polysaccharides. Compensatory changes in matrix polysaccharide abundance, interactions, and/or arrangements might arise in the absence of sufficient cellulose and cause this mechanical difference. Alternatively, a reduction in cellulose density might provide more contact sites at which matrix polymers can interact with CMFs, with these cellulose-matrix interactions perhaps being mechanically stronger than cellulose-cellulose bundling interactions. Such increases in interactions between linker elements and CMFs should increase wall stiffness (Nili et al., 2015). This idea is further supported by the fact that shear moduli (*G*_12_, *G*_23_, and *G*_23_) in *cesa3*^*je*5^ FEMs became isotropic. Together, these data suggest that reversible deformations of matrix polymers that interact with CMFs, in addition to CMF rearrangements (Rui and Anderson, 2016), occur during longitudinal deformation in guard cell walls.

Xyloglucan is thought to act as a tether or spacer between CMFs (Cosgrove, 2015; Park and Cosgrove, 2015), and because CMFs are highly oriented in guard cell walls (Fujita and Wasteneys, 2014; Rui and Anderson, 2016), the loss of xyloglucan in *xxt1 xxt2* mutants might have anisotropic effects on wall stiffness. This idea is supported by the observation that *xxt1 xxt2* hypocotyls and open stomata display more anisotropic cellulose organization than wild type controls (Rui and Anderson, 2016; Xiao et al., 2016). The lower Young's moduli (*E*_1_, *E*_2_, and *E*_3_) and a reverse in the direction of anisotropy, with *E*_1_ becoming larger than *E*_2_ and *E*_3_ that we modeled in *xxt1 xxt2*, suggest that a loss of cellulose-xyloglucan interconnections might not be compensated for by other matrix polymers such as pectins. The decreased wall stiffness modeled for this xyloglucan-deficient mutant corroborates a measured increase in plastic and elastic compliance in *xxt1 xxt2* mutant petioles (Park and Cosgrove, 2012).

In *PGX1 OE* guard cells, which should have a reduction in HG molecular mass (Xiao et al., 2014; Phyo et al., 2017), the decrease in *E*_1_ and increase in *E*_2_ and *E*_3_ we modeled might be explained by an increased number of pectin fragments that can interact more extensively with other wall components, enhancing the mechanical anisotropy of the guard cell wall. The decrease in *E*_1_ in *PGX1 OE* guard cells agrees with previous data showing that promoting pectin de-methyl-esterification decreases cell wall stiffness as probed by AFM (Peaucelle et al., 2011, 2015). However, our findings of an increase in *E*_2_ and *E*_3_ in *PGX1 OE* compared to Col-0 do not agree with these previous studies, but do agree with other studies reporting stiffening effects of pectin de-methyl-esterification (Al-Qsous et al., 2004; Pelloux et al., 2007). In this case, pectin seems to play an important role in constraining the expansion of guard cell walls in circumferential vs. radial directions. Such a constraint should be an effective mechanism for anisotropic guard cell expansion during stomatal opening, over and above the radial-longitudinal constraints imposed by CMFs.

In addition, increased anisotropy upon pectin de-methyl-esterification, which can lead to either pectin crosslinking or degradation, is also observed by Peaucelle et al. (2015). A drastic increase in *G*_23_ in *PGX1 OE* further suggests a contribution of HG to the anisotropic mechanics of guard cell walls. A higher *G*_23_ in *PGX1 OE* cell walls also implies that strains in *E*_2_ and *E*_3_ are similar to one another during stomatal opening due to a higher resistance to change in angular deformation between the circumferential and radial directions than between other pairs of directions. Considering that shear modulus represents mechanical resistance to shape changes via interactions between layers, reducing HG molecular mass seems to tighten the mechanical coordination between circumferential and radial directions. In addition, decreased HG molecular mass resulted in decreased shear modulus *G*_12_, which can be attributed to decreased inter-lamellar crosslinking in *PGX1 OE* guard cells. Overall, the decrease in cell wall stiffness probed by AFM (Peaucelle et al., 2011, 2015) suggests that *E*_1_ plays a more crucial role than *E*_*2*_ and *E*_*3*_ in determining overall stiffness.

### Guard cells are constrained by neighboring cells during stomatal opening

To achieve agreement between our experimental observations and modeling results, we considered mechanical constraints from neighboring cells. This concept differs from scenarios invoking mechanical advantages between pavement cells and guard cells (Meidner and Mansfield, 1968; Aylor et al., 1973; DeMichele and Sharpe, 1973; Edwards et al., 1976; Zeiger et al., 1987; Niklas, 1992; Franks et al., 1998). We found that physical constraints from neighboring cells limit deformations of guard cell width and stomatal complex length when stomata open. In other words, this observation suggests that increased turgor alone cannot induce the experimentally observed stomatal opening with almost no radial expansion of guard cells or changes in stomatal complex length (Table 1 and Figure 7).

We and others (Rui and Anderson, 2016; Woolfenden et al., 2017) have observed very little or no change in guard cell width upon stomatal opening. In our FEMs, none of the physically possible combinations of wall mechanical properties resulted in as small a change in guard cell width as in the experimental measurements (Figure 7), with the exception of *xxt1 xxt2*. This discrepancy between experimental measurements and modeling results strongly suggests that there may be specific mechanical properties that constrain the deformation of the ventral, or pore, side of the guard cell. One possible origin of this constraint is the enhanced thickening of the inner and outer periclinal walls in proximity to the ventral plane (Supplemental Figure 1), which would allow for stretching but not significant lateral displacement of the ventral wall itself with respect to the guard cell radius.

For stomatal complex length, there are marginal changes in Col-0 upon stomatal opening, whereas it actually decreased in *cesa3*^*je*5^, *xxt1 xxt2*, and *PGX1 OE* plants. Such reductions can only be achieved by applying additional external forces, because higher mechanical stiffness can only achieve smaller deformation but not a deformation in the opposite direction to the imposed turgor.

An extracellular load applied to the junction area during stomatal opening would prevent the elongation of the whole stomatal complex, which is consistent with a recently proposed polar fixing model (Carter et al., 2017). Combining FEM results of higher stress near stomatal junctions with the additional mechanical interaction with pavement cells in the same region, an enrichment of pectic polysaccharides near guard cell junctions (Carter et al., 2017; Rui et al., 2017) can be hypothesized to contribute to the enhanced mechanical stiffness in this region. On the other hand, a higher level of stress near periclinal ridge regions seems to be linked to thicker guard cell walls in this region. However, an additional constraint at the junction was not required for the mutant genotypes we analyzed, especially in the case of *xxt1 xxt2*, suggesting that some cell wall defects might lead to mechanical uncoupling of stomatal complexes from their epidermal neighbors.

### Future work

Even though modeled geometries of guard cells of different genotypes closely matched measurements from 3D microscopic images of actual stomata, our current FEMs could be further improved by higher-resolution microscopy and automated landmarking. Automated landmarking to trace the innermost and/or outermost layers of guard cell walls in 3D will allow FE modeling to incorporate guard cell wall properties such as varying thickness around the periphery of each guard cell (Zhao and Sack, 1999) and to further investigate comprehensive stomatal geometries such as stomatal pore area. Expansion of the automated landmarking approach will also allow neighboring cells to be modeled together so that their interaction with guard cells can be simulated. Furthermore, a multiscale computational model that describes individual components in the walls of guard cells and their molecular interactions with high spatial precision could be used to further examine how plants regulate guard cell walls to achieve repetitive and elastic deformations during stomatal dynamics.

## Accession numbers

Sequence data from this article can be found in the Arabidopsis Genome Initiative or GenBank/EMBL databases under the following accession numbers: *CESA3* (At5g05170), *XXT1* (At3g62720), *XXT2* (At4g02500), and *PGX1* (At3g26610).

## Author contributions

YR and CA designed experiments. YR performed experiments. BK and JW built computational analysis pipelines for imaging data. HY and VP developed FE models. HY, VP, YR, BK, JW, and CA analyzed results and HY, YR, BK, VP, JW, and CA wrote the manuscript.

### Conflict of interest statement

The authors declare that the research was conducted in the absence of any commercial or financial relationships that could be construed as a potential conflict of interest.
