# Supplementary material for: Mechanical Effects of Cellulose, Xyloglucan, and Pectins on Stomatal Guard Cells of Arabidopsis thaliana
Source: Front Plant Sci. 2018 Nov 5;9:1566. doi: 10.3389/fpls.2018.01566 (PMC6230562; doi:10.3389/fpls.2018.01566)
Supplement: Supplementary file 1 [file Table_1.pdf]

Supplemental Table 1. Estimates of Guard Cell Wall Thickness in Col-0, *cesa3^{je5}*, *xxt1 xxt2*, and *PGX1 OE* plants.

	Lower periclinal wall thickness (µm)	Upper periclinal wall thickness at cuticular ledges (µm)	Upper periclinal wall thickness away from cuticular ledges (µm)	Ventral wall thickness (µm)	Dorsal wall thickness (µm)
Col-0	2.2 ± 0.1^{a}	1.4 ± 0.0^{a}	0.6 ± 0.0^{a}	0.8 ± 0.0^{a}	0.4 ± 0.0^{a}
cesa3^{je5}	2.6 ± 0.1^{b}	1.5 ± 0.1^{a}	0.7 ± 0.0^{b}	0.7 ± 0.0^{b}	0.4 ± 0.0^{b}
xxt1 xxt2	2.3 ± 0.1^{a}	1.4 ± 0.0^{a}	0.7 ± 0.0^{ab}	0.8 ± 0.0^{ab}	0.4 ± 0.0^{bc}
PGX1 OE	2.6 ± 0.1^{b}	1.7 ± 0.0^{b}	0.7 ± 0.0^{ab}	0.8 ± 0.0^{a}	0.3 ± 0.0^{c}

Wall thickness was measured at five different regions for a given guard cell. Values are presented as mean ± SE ($n \geq 53$ stomata from 4 individual plants for each genotype). Lowercase letters represent significantly different groups ($P < 0.05$, one-way ANOVA and Tukey test).
