# Supplementary material for: Mechanical Effects of Cellulose, Xyloglucan, and Pectins on Stomatal Guard Cells of Arabidopsis thaliana
Source: Front Plant Sci. 2018 Nov 5;9:1566. doi: 10.3389/fpls.2018.01566 (PMC6230562; doi:10.3389/fpls.2018.01566)
Supplement: Supplementary file 2 [file Table_2.pdf]

Supplemental Table 2. Measurement of stomatal pore dimensions, guard cell pair dimensions, and guard cell geometry in FE models of wild type (Col-0), *cesa3^{je5}*, *xxt1 xxt2*, and *PGX1 OE* stomata.

Treatment	Genotype	Avg stomatal pore width (µm)	Avg stomatal pore length (µm)	Avg aspect ratio of pores	Avg pore area (µm^2)	Avg guard cell width (µm)	Avg guard cell arc length (µm)
Closed	Col-0	1.2 ± 0.3^a	11.9 ± 1.0^a	0.10 ± 0.03^a	14.3 ± 3.1^a	6.5 ± 0.1^a	20.7 ± 1.1^a
	cesa3^{je5}	2.5 ± 0.2^b	11.2 ± 0.5^a	0.23 ±0.02^b	27.4 ± 1.4^b	6.2 ± 0.1^a	22.6 ± 0.6^b
	xxt1 xxt2	0.6 ± 0.2^a	12.2 ± 0.5^a	0.04 ±0.02^a	9.1 ± 2.9^a	6.3 ± 0.1^a	19.7 ± 0.8^{ac}
	PGX1 OE	0.7 ± 0.2^a	12.4 ± 0.6^a	0.05 ±0.02^a	19.7 ± 3.2^b	5.9 ± 0.1^a	22.2 ± 0.7^{bc}
Open	Col-0	3.1 ± 0.3^a	13.7 ± 1.2^a	0.34 ± 0.03^a	28.1 ± 3.8^a	6.8 ± 0.1^a	23.0 ± 1.2^a
	cesa3^{je5}	4.6 ± 0.2^b	13.3 ± 0.4^a	0.42 ± 0.02^b	43.2 ± 1.9^b	6.8 ± 0.1^a	24.3 ± 0.8^a
	xxt1 xxt2	1.6 ± 0.3^c	14.0 ± 0.6^a	0.14 ± 0.02^c	18.9 ± 3.5^a	6.2 ± 0.1^a	23.6 ± 1.3^b
	PGX1 OE	3.8 ± 0.3^a	15.2 ± 0.8^a	0.28 ± 0.01^a	51.2 ± 4.6^b	6.8 ± 0.1^a	24.3 ± 0.9^a

Values are presented as mean ± SE (n = 10 stomata per genotype per treatment from two independent experiments). Lowercase letters represent significantly different groups ($P < 0.05$, one-way ANOVA and Tukey test; ANOVA was performed within each treatment).

Stomatal complex length and width are not listed here because they were used as a prescribed boundary condition in the stomatal FE models to calculate interactions between guard cells and pavement cells. As a result, stomatal complex length and width were exactly same as the experimental measurement. Therefore, they are not listed here.
