# Supplementary material for: Mechanical Effects of Cellulose, Xyloglucan, and Pectins on Stomatal Guard Cells of Arabidopsis thaliana
Source: Front Plant Sci. 2018 Nov 5;9:1566. doi: 10.3389/fpls.2018.01566 (PMC6230562; doi:10.3389/fpls.2018.01566)
Supplement: Supplementary file 5 [file Image_1.pdf]

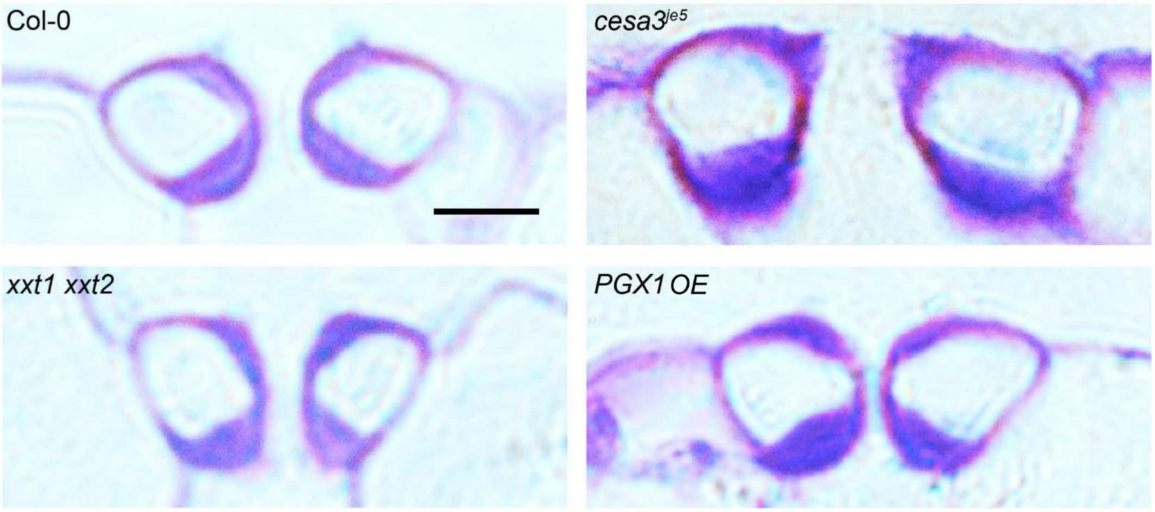

Supplemental Figure 1. Representative toluidine blue staining images of cross sections of Col-0, *cesa3*^{je5}, *xxt1 xxt2*, and *PGX1 OE* stomata from 3- to 4-week-old rosette leaves. Scale bar is 5 µm. See also Supplemental Table 1 for measurements of guard cell wall thickness from these images.
