# Supplementary material for: Mechanical Effects of Cellulose, Xyloglucan, and Pectins on Stomatal Guard Cells of Arabidopsis thaliana
Source: Front Plant Sci. 2018 Nov 5;9:1566. doi: 10.3389/fpls.2018.01566 (PMC6230562; doi:10.3389/fpls.2018.01566)
Supplement: Supplementary file 6 [file Image_2.pdf]

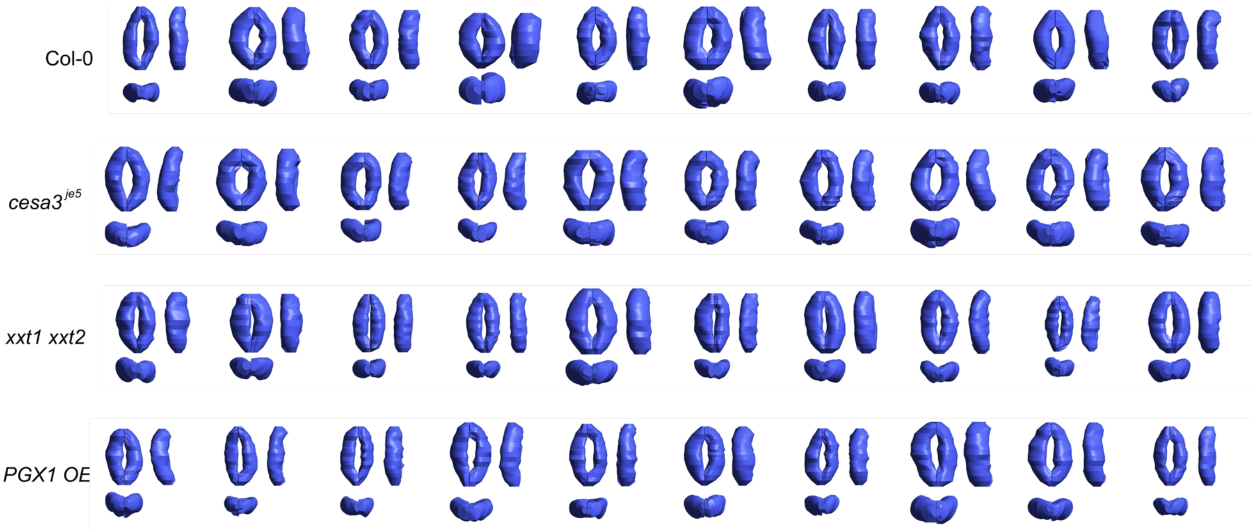


**Supplemental Figure 2.** Representative surface model of stomatal guard cell of Col-0, *cesa3*$^{je5}$, *xxt1 xxt2*, and *PGX1 OE* genotypes. Surface representation of guard cells were used in FE models. Shown are the projected views of each model on XY (center), YZ (left), and XZ (bottom) planes.
