# Supplementary material for: Mechanical Effects of Cellulose, Xyloglucan, and Pectins on Stomatal Guard Cells of Arabidopsis thaliana
Source: Front Plant Sci. 2018 Nov 5;9:1566. doi: 10.3389/fpls.2018.01566 (PMC6230562; doi:10.3389/fpls.2018.01566)
Supplement: Supplementary file 7 [file Image_3.pdf]

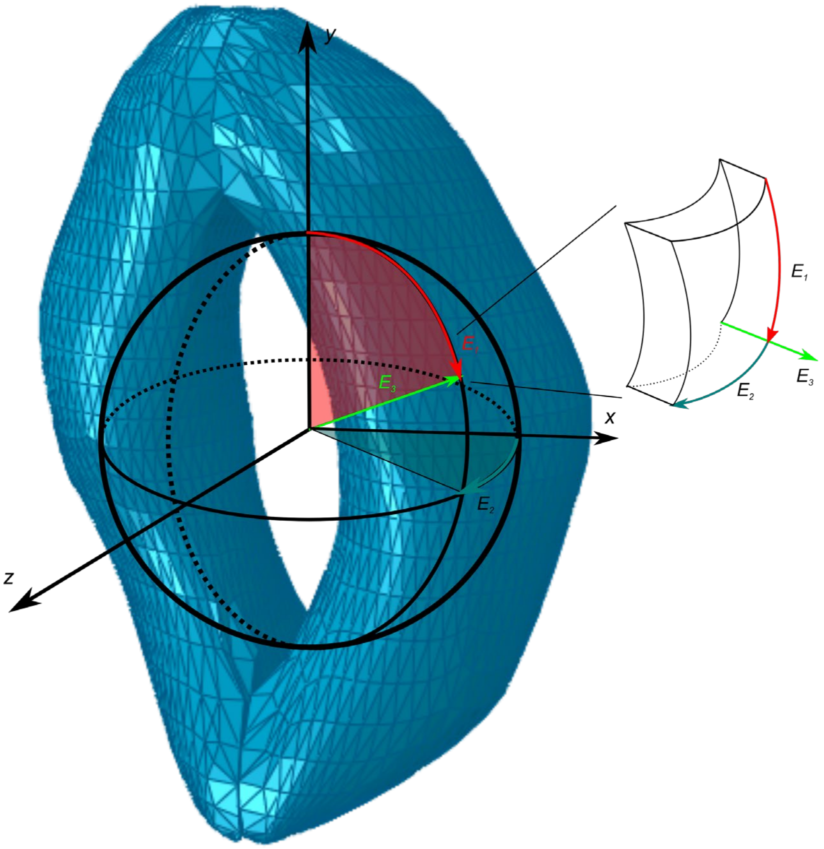

Supplemental Figure 3. Spherical coordinates used for the assignment of material properties for shell elements in FEMs. E_1 is in the longitudinal direction of the guard cell, E_2 is in the circumferential direction, and E_3 is in the radial direction, which represents the direction of wall thickness. FE models shown here has been processed with a Laplacian smoothing (Vollmer et al. 1999). Results of stomatal opening using original and smoothed FE models are identical.
