# Supplementary material for: Mechanical Effects of Cellulose, Xyloglucan, and Pectins on Stomatal Guard Cells of Arabidopsis thaliana
Source: Front Plant Sci. 2018 Nov 5;9:1566. doi: 10.3389/fpls.2018.01566 (PMC6230562; doi:10.3389/fpls.2018.01566)
Supplement: Supplementary file 8 [file Image_4.pdf]

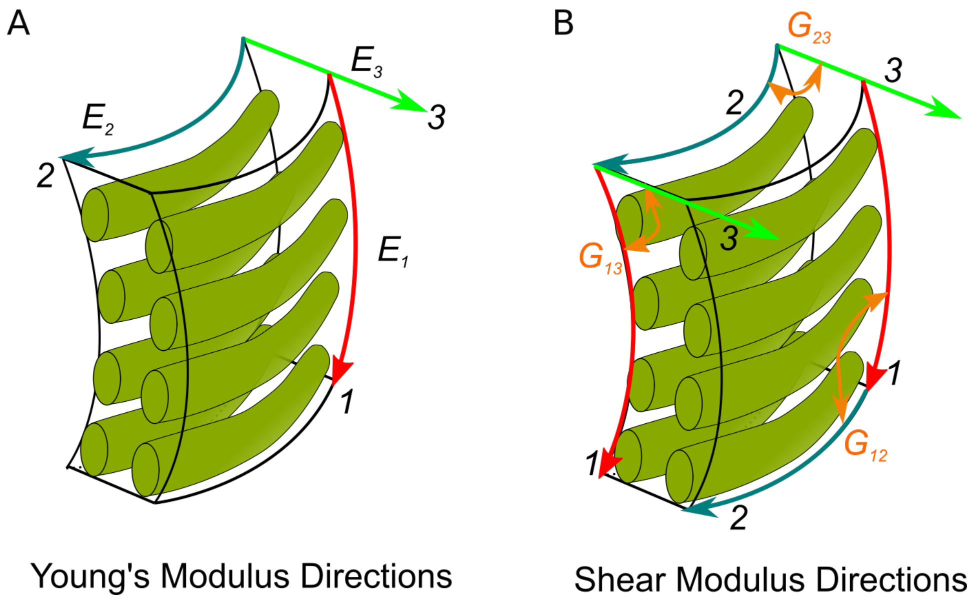


**Supplemental Figure 4.** Directions of elastic moduli including Young's modulus and shear modulus. Green circular cylinders represent cellulose microfibrils whose major direction coincides with the circumferential axis denoted by 2. A. Directions of Young's modulus coincide with the respective axes. B. Shear modulus lies on a plane consisting of two respective axes. $G_{12}$ acts on the plane consisting of the longitudinal axis and the circumferential axis. $G_{13}$ acts on the plane consisting of the longitudinal axis and the radial axis. $G_{23}$ acts on the plane consisting of the circumferential axis and the radial axis.
