# Supplementary material for: Mechanical Effects of Cellulose, Xyloglucan, and Pectins on Stomatal Guard Cells of Arabidopsis thaliana
Source: Front Plant Sci. 2018 Nov 5;9:1566. doi: 10.3389/fpls.2018.01566 (PMC6230562; doi:10.3389/fpls.2018.01566)
Supplement: Supplementary file 9 [file Image_5.pdf]

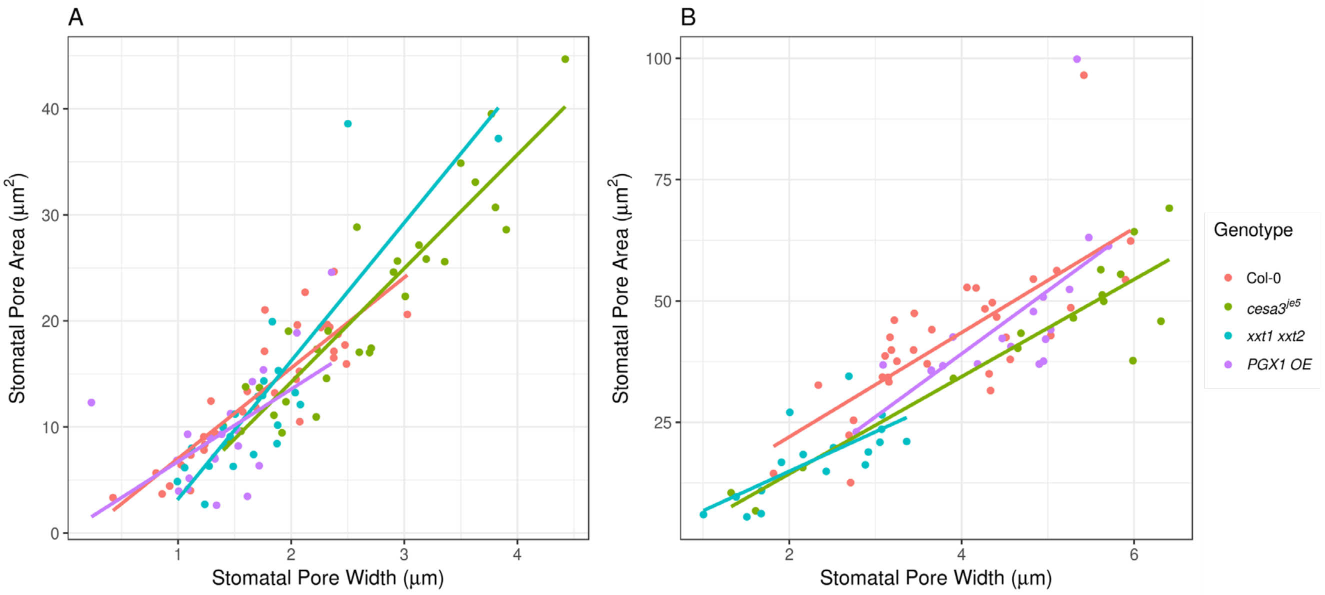

Supplemental Figure 5. Stomatal pore area and width are linearly correlated in the closed (A) or open (B) state. For measured pore areas in the closed state, n = 36 stomata for Col-0, 32 for *cesa3^{je5}*, 39 for *xxt1 xxt2*, and 34 for *PGX1 OE*, respectively. For measured pore area in the open state, n =33 stomata for Col-0, 15 for *cesa3^{je5}*, 17 for *xxt1 xxt2*, and 19 for *PGX1 OE*, respectively. Trend lines represent linear regression models for each genotype and stomatal state, respectively. For closed stomata, the coefficients of pore width for Col-0, *cesa3^{je5}*, *xxt1 xxt2*, and *PGX1 OE* are 8.5 (R^2 = 0.77), 10.7 (R^2=0.85), 13.1 (R^2=0.72), and 6.8 (R^2 = 0.30), respectively. For open stomata, the coefficients of pore width for Col-0, *cesa3^{je5}*, *xxt1 xxt2*, and *PGX1 OE* are 10.7 (R^2 = 0.55), 10.0 (R^2=0.86), 8.1 (R^2=0.50), and 13.0 (R^2 = 0.44), respectively.
